# A review of MXene-based sonocatalytic degradation for water purification^☆^

**DOI:** 10.1016/j.ultsonch.2026.108018

**Published:** 2026-08-22

**Authors:** Hak-Hyeon Kim, Seong-Nam Nam, Chang Min Park, Min Jang, Shane A. Snyder, Byung-Moon Jun, Yeomin Yoon

**Affiliations:** a Department of Environmental Science & Engineering, Ewha Womans University, 52 Ewhayeodae-gil, Seodaemun-gu, Seoul 03760, the Republic of Korea; b Department of Chemical and Environmental Science, Korea Army Academy at Yeongcheon, 495 Hoguk-ro, Gogyeong-myeon, Yeongcheon-si, Gyeongsangbuk-do 38900, the Republic of Korea; c Department of Environmental Engineering, Kyungpook National University, 80 Daehak-ro, Buk-gu, Daegu 41566, the Republic of Korea; d Department of Environmental Engineering, Kwangwoon University, 447-1 Wolgye-dong Nowon-gu, Seoul, the Republic of Korea; e School of Civil and Environmental Engineering, College of Engineering, Georgia Institute of Technology, 311 Ferst Dr NW, Atlanta, GA 30332, United States; f Department of Environmental Science and Engineering, Kyung Hee University, 1732 Deogyeong-daero, Giheung-gu, Yongin-si, Gyeonggi-do 17104, the Republic of Korea

**Keywords:** Sonocatalysis, MXenes, Degradation mechanisms, Stability, Water treatment

## Abstract

MXene-based sonocatalysis is an emerging advanced oxidation process for removing recalcitrant dyes, endocrine-disrupting compounds, and pharmaceuticals from water, where conventional treatment may achieve very limited removal by coagulation and chlorination. This review was motivated by the need to clarify how MXene structure, water quality, and ultrasound conditions control contaminant degradation, mineralization, stability, and practical applicability. We hypothesized that MXenes enhance sonochemical oxidation by promoting pollutant adsorption, cavitation, charge separation, and reactive oxygen species generation, particularly in engineered heterostructures. Recent studies were critically analyzed with respect to MXene composition and surface chemistry, ultrasound frequency and power, water-matrix effects, degradation mechanisms, hybrid sonophotocatalytic/sono-Fenton processes, mineralization, and catalyst reusability. MXene-based systems achieved up to >99% antibiotic removal, approximately 70% acetaminophen mineralization, and 80% rhodamine B degradation in 20 min; ^•^OH and O_2_^•−^ predominantly governed oxidation, while several catalysts retained more than 80–90% removal over five cycles and exhibited limited leaching. Overall, MXenes provide a versatile platform for efficient water purification by integrating conductive, adsorptive, cavitation-nucleating, and interfacial charge-transfer functions, although real-water validation, standardized evaluation, energy-efficient reactor scale-up, and toxicity assessment remain necessary for environmental deployment.

## 1 Introduction

Water is essential for all life and has historically supported the development of human civilization [1]. However, the stringent regulation of water contaminants and the sustainable management of aquatic resources have now become critical environmental and public health priorities [2]. Anthropogenic activities have continuously introduced a heterogeneous array of xenobiotics into aquatic systems [3]. Yoon et al. specifically documented the occurrence of endocrine-disrupting compounds (EDCs), pharmaceutically active compounds (PhACs), and personal care products in the Han River, highlighting the environmental relevance of these micropollutants in urban waters [4]. Capasso et al. focused on humic substances as representative natural organic matter (NOM), emphasizing their importance in contaminant transport and removal behavior [5].

Globally, approximately 0.7–1.0 million metric tons of synthetic dyes are produced annually [6]. During textile dyeing processes, an estimated 10–20% of the applied dyes are not fixed to fibers and are discharged in wastewater effluents [7]. The global pharmaceutical market utilizes more than 4,000 distinct active pharmaceutical ingredients, with the annual consumption of high-demand classes such as antibiotics and analgesics estimated to exceed 100,000 metric tons worldwide [8]. This substantial usage results in the continuous introduction of bioactive, pseudo-persistent compounds into aquatic environments, primarily through human and veterinary excretion, as well as the improper disposal of unused medications [9].

Conventional water and wastewater treatment plants were designed primarily to remove turbidity, bulk organic matter, and nutrients [10]. They often do not completely remove recalcitrant dyes, EDCs, and PhACs because these compounds are present at low concentrations and are difficult to biodegrade. Previous studies have revealed significant limitations in conventional unit operations. Westerhoff et al. showed that simulated drinking-water treatment processes incompletely removed EDCs and PhACs (approximately <25% by coagulation and <10% by chlorination) [11]. Verma et al. reviewed coagulation/flocculation technologies for textile wastewater and reported that color removal can be strongly limited depending on dye chemistry and process conditions [12]. Addressing this technological gap requires the implementation of tertiary or advanced treatment processes capable of higher selectivity and enhanced degradation potential. Promising advanced technologies currently under investigation include physical separation methods such as reverse osmosis [13], nanofiltration [14], and various emerging nanoadsorbents (e.g., carbon nanotubes [15], graphene oxide [16], metal–organic frameworks (MOFs) [17], and MXenes [18]), as well as oxidation/reduction techniques (e.g., plasma oxidation [19], ultraviolet irradiation/photolysis [20], [21], ozonation [22], and sonodegradation [23]).

Sonodegradation, which leverages the physical and chemical effects of acoustic cavitation, is an advanced oxidation process that has gained significant attention for the treatment of recalcitrant organic pollutants in water [24]. It has been effectively applied to oxidize a broad spectrum of organic contaminants, offering relatively simple operation, rapid treatment, and a favorable environmental profile [25]. The underlying mechanism proceeds through three stages: (i) cavitation bubble formation induced by high-frequency sound waves; (ii) bubble growth accompanied by extreme localized conditions (temperatures up to approximately 5,000 K and pressures near 1,000 atm); and (iii) violent bubble collapse, which promotes water sonolysis and the generation of highly reactive ^•^OH and O_2_^•−^ radicals [26]. To further enhance sonochemical oxidation efficiency, sonocatalytic processes have been developed using various (nano)catalysts, such as carbon nanotubes [27], biochar [28], graphene oxide [29], metal–organic frameworks [30], and MXenes [31].

In particular, MXenes offer several advantages as sonocatalysts for water treatment. The two-dimensional layered structure of MXenes provides a high specific surface area. Their –O, –OH, and –F terminations promote pollutant adsorption and increase the availability of active sites [32]. Their high electrical conductivity promotes rapid charge transfer and suppresses electron/hole (e^−^/h^+^) recombination when ultrasonication is combined with photo- or electro-processes, thereby enhancing radical generation (e.g., ^•^OH and O_2_^•−^) [33]. Strong interfacial interactions with cavitation bubbles improve hot-spot utilization and accelerate the formation of sono-generated reactive species [34]. MXenes also exhibit tunable surface chemistry (via etching or post-treatment), enabling optimization of hydrophilicity, stability, and catalytic activity in aqueous media [18]. Furthermore, their good dispersibility and reusability, along with the possibility of magnetic or composite designs, support practical recovery and long-term operation [35].

Current reviews on MXene-based sonocatalytic and photocatalytic systems have provided important but distinct perspectives. In particular, Vasseghian et al. emphasized MXenes as heterogeneous sonocatalysts for pollutant degradation [33], while Zhong et al. focused mainly on MXene-based and MXene-derived photocatalysts, including band-structure engineering and charge transport [34]. In addition, Thaha and Sathishkumar reviewed MXene nanoarchitectures under sonophotocatalytic environments, with emphasis on recent catalyst designs [35]. Despite the growing interest in MXene-enabled sonocatalysis, a comprehensive and critical synthesis of their structure–activity relationships, operational performance, and degradation mechanisms remains limited. Therefore, this review provides a rigorous and systematic evaluation of MXene-based sonocatalysts for the ultrasonic degradation of selected textile dyes, EDCs, and PhACs. In particular, it aims to: (i) establish how MXene composition, surface terminations, interlayer architecture, morphology, and heterointerface engineering with supporting materials regulate radical generation, charge separation, and sonocatalytic activity; (ii) elucidate the roles of solution chemistry and reaction environment, including pH, inorganic ions, NOM, promoters/scavengers, cavitation dynamics, degradation intermediates, transformation pathways, and mineralization efficiency; and (iii) identify optimal sonochemical operating windows, including ultrasonic frequency, acoustic power density, catalyst dosage, and process intensification through hybrid systems such as US/UV, US/Fenton, and other advanced oxidation configurations. Additionally, a prospective analysis of future research directions is presented, highlighting strategic pathways to overcome current technical barriers.

## 2 US treatment performance

### 2.1 Degradation affected by water quality and sonication conditions

Water quality and sonication conditions critically govern degradation pathways and the lifetime of MXene-based sonocatalysts [33]. In particular, pH, specific ions (Cl^−^, F^−^, and transition metals), ionic strength, background organics, and temperature can significantly influence sonocatalytic activity [36]. Sonication parameters also influence cavitation intensity, reactive oxygen species (ROS) generation, and mechanical shear. Changes in frequency, power, or duration can therefore produce outcomes ranging from mild surface modification to rapid fragmentation and complete oxide conversion [37].

#### 2.1.1 Effects of pH

Solution pH critically governs sonocatalytic performance by altering the catalyst surface charge (relative to the point of zero charge, pH_pzc_). This, in turn, affects contaminant adsorption, contaminant speciation/ionization and reactivity, cavitation bubble chemistry and collapse intensity, the generation and lifetime of dominant ROS (^•^OH vs O_2_^•−^), and electron–hole (e^−^/h^+^) recombination and charge transfer [23]. Therefore, the optimal pH depends on the catalyst, contaminant, and water matrix, as these factors collectively shift reaction pathways and degradation efficiency [26].

**Fig. 1:**
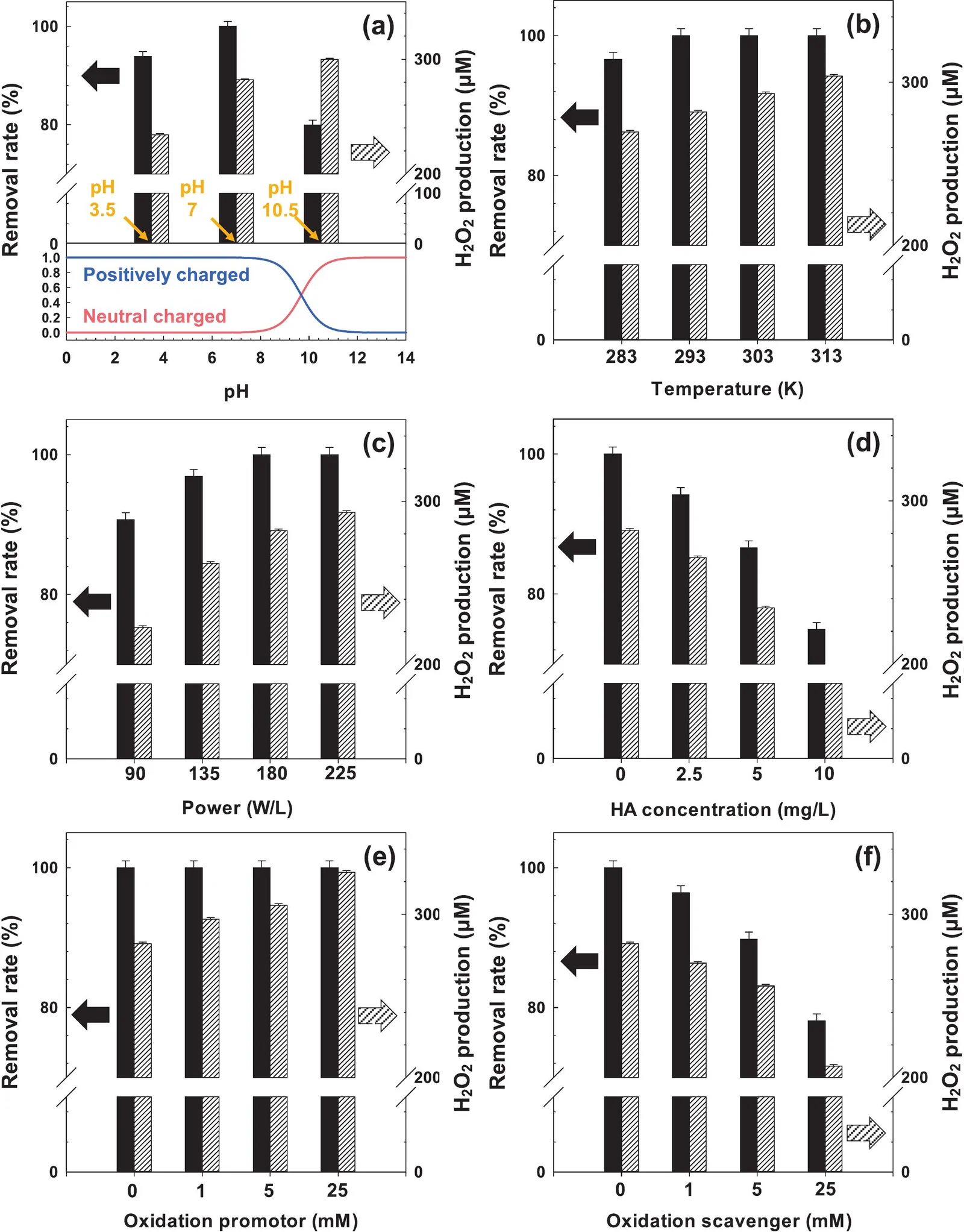
Effect of (a) pH, (b) temperature, (c) power intensity, (d) humic acid, (e) oxidation promoter (CCl_4_), and (f) oxidation scavenger (methanol) on US performance in the presence of multi-layered Ti_3_C_2_T_x_ performance. Operating conditions: initial [verapamil] = 5 μM; Ti_3_C_2_T_x_ = 45 mg/L; reaction time = 60 min [38].

Multi-layered Ti_3_C_2_T_x_ MXenes were evaluated as both catalysts and adsorbents for the removal of verapamil (a non-dihydropyridine calcium channel blocker) under various pH conditions during US treatment [38]. Fig. 1a shows that H_2_O_2_ production increased continuously from pH 3.5 to 10, while verapamil removal varied with pH (94% at pH 3.5, >99% at pH 7, and 80% at pH 10.5). At low pH, the concentration of free ^•^OH is higher due to enhanced H_2_O_2_ decomposition. These free radicals preferentially oxidize verapamil rather than recombining to form H_2_O_2_ [39]. In contrast, at pH 10.5, the higher measured H_2_O_2_ concentration is attributed to faster ^•^OH recombination relative to ^•^OH reaction with verapamil [40].

The speciation of verapamil also varies with pH according to its basic pK_a_ (9.68). Below the pK_a_, verapamil is predominantly protonated and positively charged, promoting electrostatic attraction to the negatively charged Ti_3_C_2_T_x_ surface and favoring adsorption-driven removal. However, under the present US treatment conditions with Ti_3_C_2_T_x_ the dominant removal mechanism is oxidative degradation rather than adsorption. Although verapamil speciation at pH 3.5 and pH 7 is similar, the greater H_2_O_2_ production observed at pH 7 leads to more extensive ^•^OH-mediated oxidation and, consequently, higher removal efficiency [38].

**Fig. 2:**
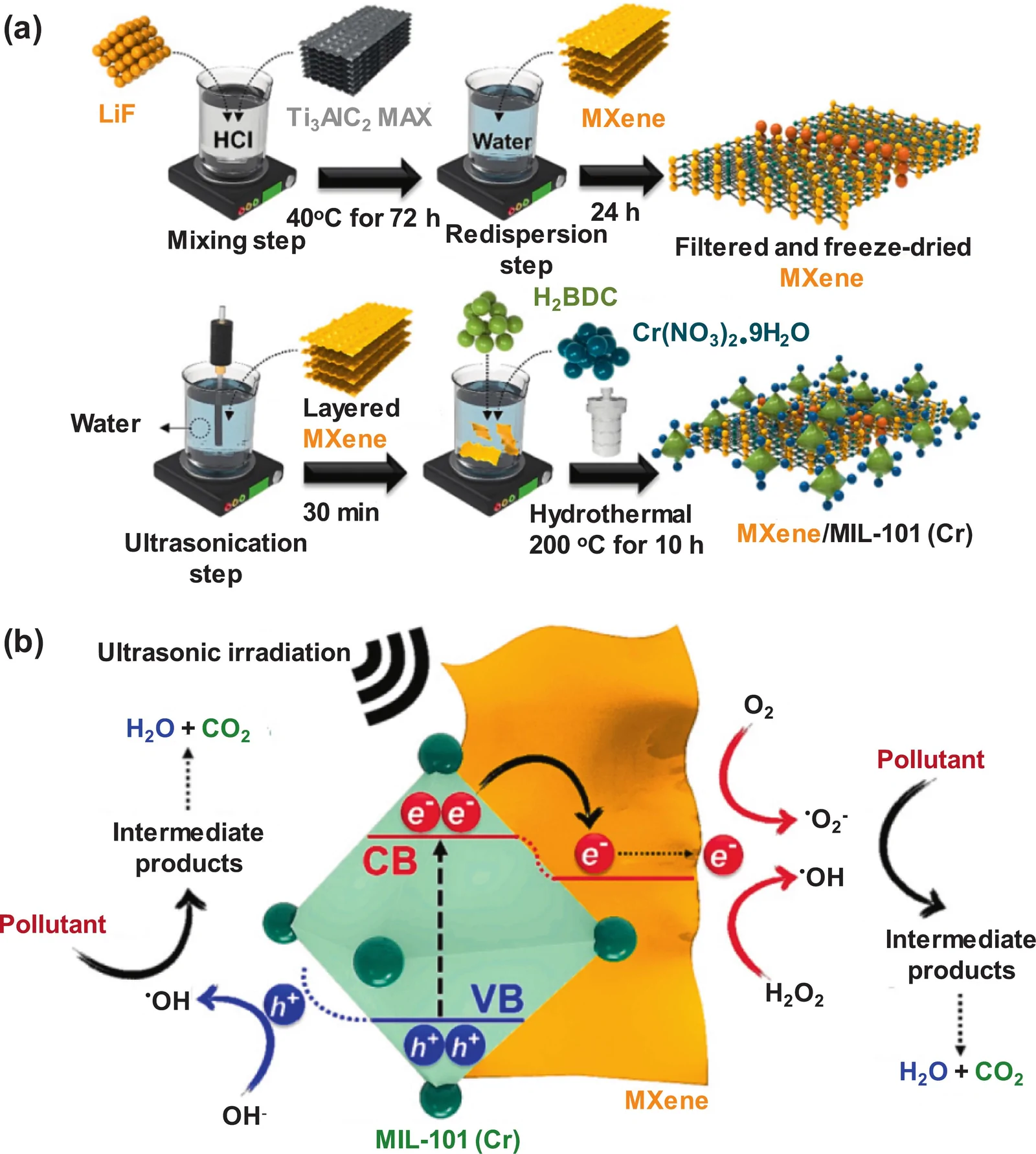
(a) Schematic depicting the synthesis of Ti_3_C_2_T_x_/MIL-101(Cr). (b) Putative mechanism for the sonocatalytic decomposition of acetaminophen and sulfadiazine by MXene/MIL-101(Cr) in an aqueous solution [41].

Various mass loadings of Ti_3_C_2_T_x_-coupled MIL-101(Cr) (Ti_3_C_2_T_x_-MIL) nanocomposites were synthesized via a hydrothermal route to evaluate their potential as sonocatalysts for acetaminophen degradation in water [41]. A schematic of the fabrication procedure is shown in Fig. 2a. Across the pH range of 3–11, the sonocatalyst exhibited superior performance under near-neutral conditions. The pH_pzc_ of Ti_3_C_2_T_x_-MIL was 7.9, reflecting the large specific surface areas of both MXene and MOF components. Under acidic conditions (pH < 6), H_2_O_2_ readily decomposes to produce ^•^OH, favoring acetaminophen oxidation. Although H_2_O_2_ formation was higher at alkaline pH, rapid ^•^OH recombination limited its effective utilization [42]. At pH > 8, CO_2_ formation further scavenges ^•^OH and suppresses catalytic activity.

In addition, pH influences acetaminophen speciation and surface interactions: when pH < pH_pzc_ the catalyst surface is negatively charged and electrostatically attracts protonated (positively charged) acetaminophen, enhancing adsorption. Nevertheless, the observed maximum sonocatalytic efficiency at neutral pH indicates that ultrasonically driven oxidative degradation dominates removal. Furthermore, the strong water adsorption capacity of MIL likely promotes interactions between dissolved acetaminophen and surface-generated ROS, thereby accelerating degradation [43]. The superior catalytic performance at neutral pH compared with acidic conditions can therefore be attributed to increased H_2_O_2_ production and more favorable ROS-mediated oxidation under these conditions [41].

One-dimensional (1D) CoTiO_3_ microrods were uniformly decorated on two-dimensional (2D) Ti_3_C_2_T_x_ MXene nanosheets via a liquid self-assembly technique to produce CoTiO_3_@Ti_3_C_2_T_x_, which was investigated as an efficient sonocatalyst for the degradation of bisphenol A [44]. The catalyst exhibited high degradation activity across a pH range of 3.0–10.0, with maximum removal at pH 7. Degradation efficiency declined slightly under alkaline conditions (pH 8 and 10) and showed only modest variation under strongly acidic conditions (pH 3).

These trends can be explained by the acid-base properties of bisphenol A and the surface charge characteristics of the catalyst. Ti_3_C_2_T_x_ carries a net negative surface charge due to abundant functional groups (–OH, =O, and –F) [45] becomes increasingly negatively charged as pH increases [46]. Bisphenol A (pK_a_ ≈ 10.2) remains predominantly neutral under mildly acidic to neutral conditions but ionizes to the phenoxide anion near and above its pK_a_ [47]. Consequently, in the pH 5–8 range, adsorption and degradation are favored due to minimal electrostatic repulsion. Additional non-electrostatic interactions (e.g., hydrogen bonding and π–π/hydrophobic interactions) further promote substrate–catalyst association [48]. At higher pH values, increased deprotonation enhances electrostatic repulsion, thereby reducing adsorption and degradation efficiency. However, under strongly acidic conditions (pH 3), the formation of highly oxidizing ^•^OH likely compensates for reduced adsorption, sustaining appreciable degradation [44].

Solution pH critically influences sonocatalysis by modulating catalyst surface charge (relative to pH_pzc_), contaminant speciation, cavitation chemistry, ROS profiles, and charge transfer/recombination. Across MXene-based systems, near-neutral pH generally maximizes oxidative degradation over adsorption: (i) Ti_3_C_2_T_x_ achieved the highest verapamil removal at pH 7, despite higher H_2_O_2_ production under alkaline conditions, due to ^•^OH recombination at elevated pH; (ii) Ti_3_C_2_T_x_-MIL exhibited peak acetaminophen degradation near neutral pH, where improved ROS production and utilization outweigh increased ^•^OH formation under acidic conditions; and (iii) CoTiO_3_@Ti_3_C_2_T_x_ showed optimal bisphenol A degradation at pH 7, whereas deprotonation near the pK_a_ reduced adsorption affinity and degradation efficiency under alkaline conditions.

#### 2.1.2 Effects of temperature

Solution temperature affects cavitation, reaction kinetics, gas solubility, mass transfer, and radical lifetime [49]. Higher temperatures can accelerate chemical reactions but also increase vapor pressure, weaken bubble collapse, reduce dissolved O_2_, and lower ^•^OH yields. While the overall effect is system-dependent, moderate temperatures often balance enhanced kinetics with preserved cavitation intensity, resulting in optimal degradation performance [50].

Fig. 1b shows the effect of solution temperature on H_2_O_2_ production and verapamil removal in the presence of Ti_3_C_2_T_x_ [38]. H_2_O_2_ formation increased gradually with temperature, which is attributed to a lower cavitation threshold (resulting from reduced surface tension and viscosity), leading to a higher number of cavitation events and thus greater H_2_O_2_ generation at elevated temperatures [42]. Verapamil degradation was 96.6% at 283 K and reached > 99% above 293 K. The temperature dependence of the reaction rate follows the Arrhenius relationship [51]. The apparent activation energy (*E_a_*) was determined to be 1.196 kJ/mol. Such a low *E_a_* indicates a rapid transition to products and suggests diffusion-controlled behavior [40]. Increasing temperature facilitates the mass transfer of verapamil from the bulk solution into cavitation bubbles (i.e., localized zones of extreme temperatures and high ^•^OH concentration), thereby enhancing oxidative degradation [52]. However, at temperatures above the optimum, cavitation energy and collapse efficacy diminish, counteracting these positive effects and resulting in a plateau in degradation efficiency. This balance explains the observed steady verapamil degradation above 293 K [53].

The effect of temperature on the sonodegradation rates of amitriptyline and ibuprofen was evaluated at 293, 303, and 313 K in the presence of Ti_3_C_2_T_x_ [54]. The degradation rates of these PhACs increased slightly with temperature up to 303 K, but declined at 313 K. This behavior reflects the competing positive and negative effects of temperature and is consistent with trends in H_2_O_2_ production, which indirectly reflects the temperature-dependent variations in ^•^OH concentration. Solution temperature in the US process is a key parameter governing the degradation of organic contaminants, as it directly influences cavitation bubble dynamics and the consequent generation of ^•^OH [55]. Both enhancing and inhibiting effects of temperature on contaminant degradation have been reported [27], [30]. Specifically, Im et al. reported temperature-sensitive ultrasonic degradation of acetaminophen and naproxen using carbon nanotubes [27], whereas Jun et al. showed that MOF-assisted sonocatalysis also responds to temperature-dependent cavitation behavior [30]. An increase in solution temperature lowers the cavitation threshold, thereby increasing the number of cavitation bubbles; however, it also reduces cavitation intensity, resulting in lower collapse pressures [56]. Thus, the optimal temperature for achieving maximum degradation rates varies with the intrinsic properties of the target compound and applied US frequency [42]. The effect of solution temperature on the sonocatalytic degradation rate (*k*_1_) of dyes further demonstrates distinct optimal temperatures at low and high US frequencies. Moreover, the observed changes in *k*_1_ closely correlate with the amount of H_2_O_2_ generated, indicating that the degradation mechanism in this system is strongly governed by H_2_O_2_ production [57].

Solution temperature is a key parameter in sonocatalytic degradation, as it governs cavitation dynamics, radical generation, and reaction kinetics. In general, increasing temperature lowers the cavitation threshold and enhances H_2_O_2_ production and mass transfer, improving degradation of selected PhACs up to an optimal range (typically 293–303 K). At higher temperatures, weakened cavitation collapse, reduced ^•^OH production, and lower O_2_ solubility offset kinetic gains, leading to plateaued or decreased degradation. Optimal temperature is thus system- and frequency-dependent, correlating strongly with H_2_O_2_ formation.

#### 2.1.3 Effects of background common ions and NOM

Background ions, ionic strength, and NOM strongly influence sonocatalytic degradation [30]. Common anions (Cl^−^, HCO_3_^−^, SO_4_^2−^, and NO_3_^−^) and cations (Ca^2+^ and Mg^2+^) can scavenge ^•^OH or form secondary oxidants (e.g., Cl^•^/Cl_2_^•−^), buffer pH, alter ionic strength and contaminant/catalyst adsorption, and promote catalyst aggregation or surface complexation [58]. In addition, NOM (e.g., humic/fulvic substances) competes for radicals, quenches ROSs, adsorbs onto catalyst surfaces (fouling), and reduces cavitation via surface activity, thereby generally inhibiting sonocatalytic degradation [57].

The influence of humic acid and ionic strength on sonodegradation was evaluated in the presence of Ti_3_C_2_T_x_ MXene for the removal of two pharmaceutically active compounds (amitriptyline and ibuprofen) [54]. The addition of humic acid enhanced removal rates for both pharmaceuticals, consistent with Park et al. (2011), who reported that humic acid can trap ^•^OH in the presence of background ions, thereby reducing ^•^OH recombination and increasing net ^•^OH availability [59]. However, adjustment of solution conductivity using NaCl produced no significant change in sonodegradation performance, which is consistent with previous studies reporting only marginal effects of NaCl and other inorganic salts (NaHCO_3_ and Na_2_CO_3_) on ultrasound-driven decolorization of acid blue 40 [60]. These results suggest that NOM may enhance sonocatalytic degradation through radical-mediated pathways, whereas ionic strength (as NaCl) exerts minimal influence under the tested conditions [40].

As shown in Fig. 1d, the influence of NOM was further assessed on verapamil removal and H_2_O_2_ production, using humic acid as a representative NOM fraction [38]. Both verapamil removal and H_2_O_2_ yield decreased linearly with increasing humic acid concentration: >99% (no humic acid), 94.3% (2.5 mg/L), 86.6% (5 mg/L), and 74.9% (10 mg/L). This reduction is attributed to the high content of carboxylic and phenolic moieties in humic acid, which scavenge reactive radicals generated during US treatment, thereby competing with verapamil for ^•^OH and other oxidants and reducing overall oxidative capacity [61]. These findings are consistent with prior reports of NOM-mediated inhibition of advanced oxidation processes [62], [63]. In a separate study, the presence of humic acid consistently reduced sonocatalytic efficiency during acetaminophen degradation in the presence of Ti_3_C_2_T_x_-MIL [41]. This observation is attributed to competitive reactions in which the organic components of humic acid, particularly carboxylic and phenolic groups, consume free radicals that would otherwise participate in the desired catalytic degradation pathway.

**Fig. 3:**
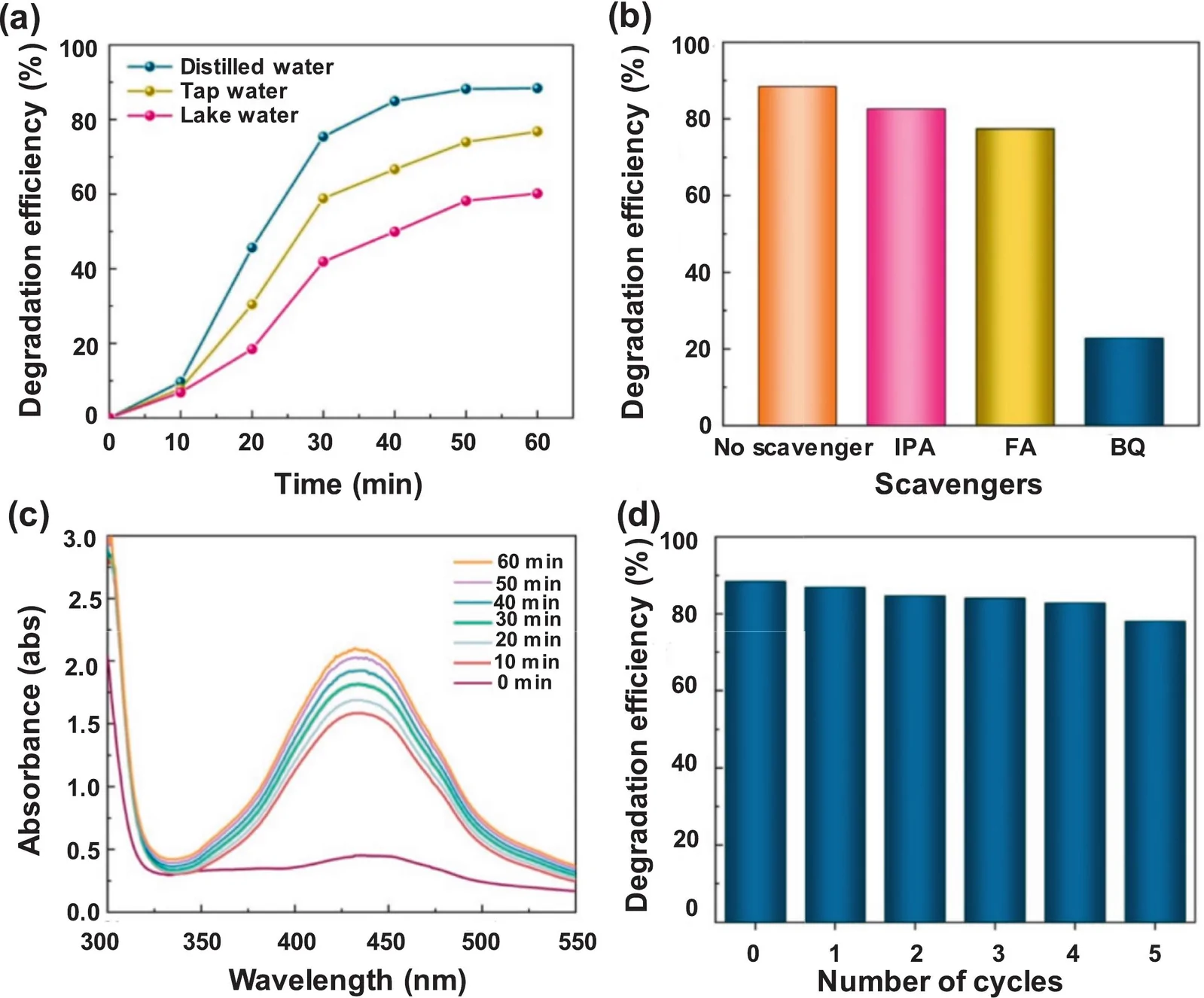
(a) Effect of water matrix, (b) effect of different scavengers on levofloxacin degradation, (c) UV–vis spectra of o–phenylenediamine-trapped ^•^OH in the US/V_4_AlC_3_ MAX phase/PMS process, and (d) reusability of V_4_AlC_3_ MAX phase (Experiment conditions: [levofloxacin] = 10 mg/L, [V_4_AlC_3_ MAX phase] = 0.5 g/L, [PMS] = 0.2 mmol/L, pH = neutral, and US power = 150 W) [64].

To evaluate the practical applicability of the V_4_AlC_3_ MAX phase/peroxymonosulfate (PMS) process, levofloxacin sonodegradation was investigated in various water matrices, including distilled water, tap water, and lake water [64]. As shown in Fig. 3a, the degradation efficiencies were 88.4%, 76.8%, and 60.2% in distilled water, tap water, and lake water, respectively. The lower efficiencies in real water samples are likely attributable to the presence of inorganic anions and NOM, which can scavenge reactive species or compete with levofloxacin for active sites on the catalyst [65]. These results demonstrate that the US-V_4_AlC_3_-PMS system remains effective in complex water matrices, underscoring its potential for practical application in the treatment of pharmaceutical contaminants.

A recent study proposed a strategy to mitigate the inefficient transfer of photoinduced e^−^ by designing an enhanced Z-scheme NiFe_2_O_4_-Bi_2_WO_6_@Ti_3_C_2_T_x_ composite with outstanding sonocatalytic activity toward ciprofloxacin degradation [66]. To evaluate its applicability in realistic wastewater conditions, ciprofloxacin degradation was examined in the presence of humic acid. The degradation efficiency decreased slightly upon addition of 1 mg/L humic acid, which can be attributed to competitive adsorption between humic acid and ciprofloxacin for the finite active sites on the catalyst surface [36]. Nevertheless, substantial degradation (>70%) was still achieved at higher humic acid concentrations (2 and 5 mg/L), highlighting the strong potential of this composite across a wide range of water matrices. In addition to NOM, common inorganic anions such as Cl^−^, NO_3_^−^, and HCO_3_^−^ can markedly affect contaminant removal during sonocatalysis. The presence of these anions (10 mM) led to different degrees of inhibition, with the degradation rate declining (approximately 5–30%) in the order Cl^−^ > NO_3_^−^ > HCO_3_^−^ [66]. The relatively weak inhibitory effect of Cl^−^ may be attributed to the formation of highly oxidizing Cl^•^ via its reaction with ^•^OH [67], which partially compensates for ^•^OH consumption. In contrast, NO_3_^−^ caused a more pronounced reduction due to its reactions with ^•^OH and e^−^ [68]. Among the anions tested, HCO_3_^−^ exhibited the strongest adverse effect due to its high scavenging capacity toward ^•^OH and h^+^ [69], resulting in substantial inhibition. Collectively, these findings indicate that anions commonly present in natural waters can compromise sonocatalytic efficiency primarily by scavenging reactive species [66].

NOM is typically one of the most destructive interferents, as it competes for adsorption sites, scavenges ^•^OH and other reactive species, suppresses interfacial electron transfer, and may contribute to catalyst fouling. HCO_3_^−^ and CO_3_^2–^ carbonate can also markedly inhibit degradation by scavenging ^•^OH and forming the less reactive CO_3_^•−^, particularly in alkaline or highly buffered waters. Cl^-^ may inhibit or redirect degradation pathways through ^•^OH scavenging and chlorine-radical formation, while phosphate can block active sites via strong surface adsorption. SO_4_^2-^ and NO_3_^–^ usually exert weaker or system-dependent effects at environmentally relevant levels, though inhibition may increase at higher concentrations. Hardness ions such as Ca^2+^ and Mg^2+^ can further affect aggregation, surface charge, and pollutant adsorption. Because inhibition thresholds depend on catalyst properties, pollutant type, pH, ultrasound conditions, dissolved oxygen, and water matrix composition, universal values are somewhat inappropriate.

#### 2.1.4 Effects of promoters and scavengers

Promoters (e.g., H_2_O_2_, persulfate, and Fenton reagents) enhance sonocatalytic degradation by increasing ROS generation, facilitating e^−^ transfer, or activating additional oxidative pathways [37]. In contrast, scavengers [e.g., isopropanol, *tert*-butanol, sodium azide, and benzoquinone (BQ)] selectively quench specific reactive species (e.g., ^•^OH, ^1^O_2_, and O_2_^•−^), thereby suppressing degradation and helping to identify dominant ROSs [54]. Both promoters and scavengers are used to tune reaction rates, probe mechanisms, distinguish radical versus non-radical pathways, and optimize additive dosing to maximize contaminant removal while minimizing side reactions [70].

**Fig. 4:**
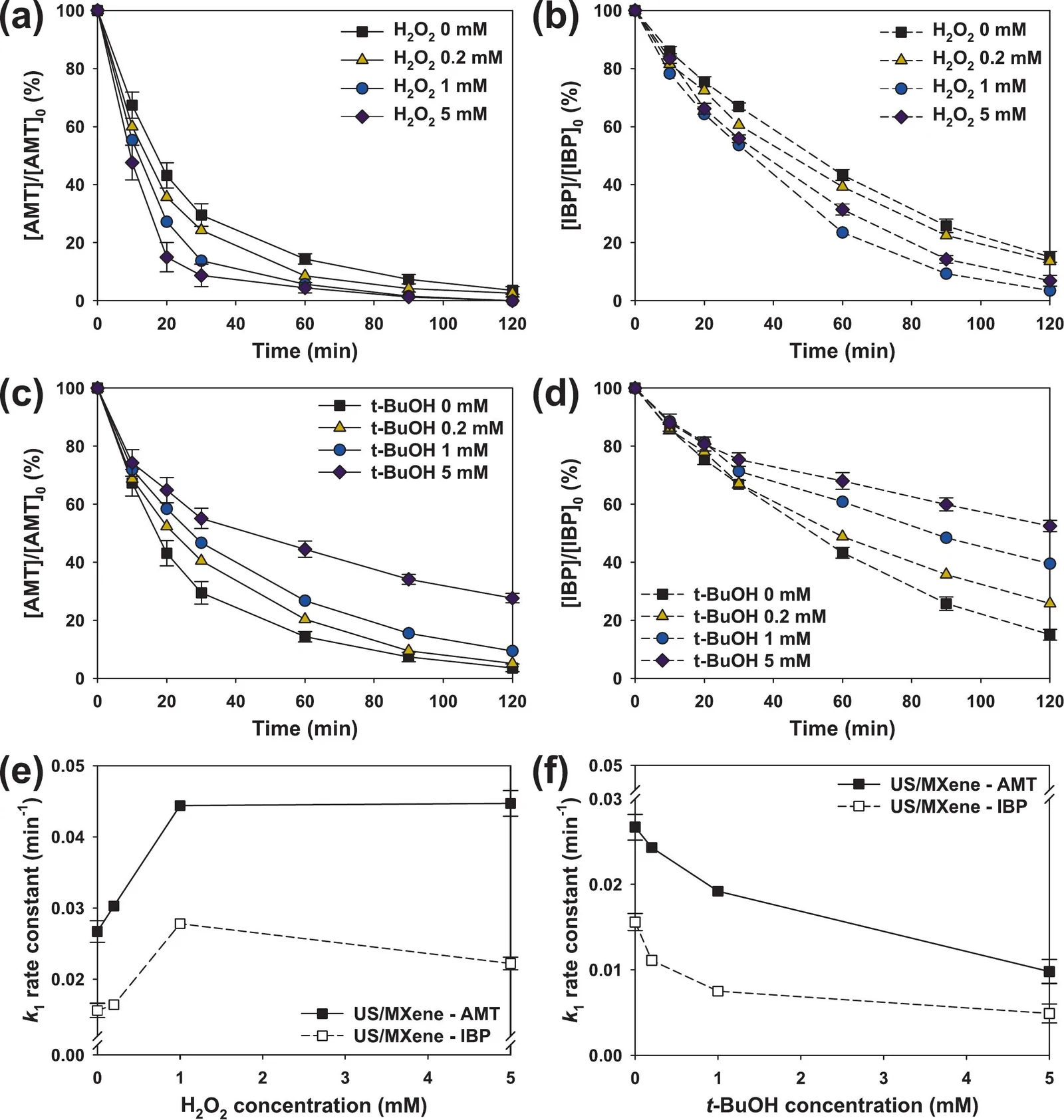
Influence of ^•^OH promoter (H_2_O_2_) on sonodegradation performance of (a) amitriptyline (AMT), (b) ibuprofen (IBP), and (e) degradation rate, and influence of ^•^OH scavenger (by tert‑butanol) on sonodegradation performance of (c) AMT, (d) IBP, and (f) degradation rate at different concentrations of ^•^OH promoter and scavenger (US/MXene system, 45 mg/L Ti_3_C_2_T_x_, 35 kHz, 50 W/L, pH 7, and 293 ± 1 K) [54].

Fig. 4a, b, and e shows the influence of the ^•^OH promoter H_2_O_2_ in the presence of Ti_3_C_2_T_x_ on sonodegradation of amitriptyline and ibuprofen [54]. Increasing H_2_O_2_ concentration up to 1 mM produced a clear increase in the apparent rate constants (*k*_1_) for both compounds, whereas further increases to 5 mM led to plateauing or slight declines in *k*_1_. Although H_2_O_2_ promotes ^•^OH formation, at relatively high concentrations it can also act as a scavenger through reactions [71], converting ^•^OH into less reactive hydroperoxyl radicals (HO_2_^•^) and ultimately H_2_O. Given the lower oxidation potential of HO_2_∙ relative to ^•^OH, the reduced *k*_1_ at 5 mM H_2_O_2_ is consistent with competitive ^•^OH consumption, supporting that ^•^OH is the primary oxidant for amitriptyline and ibuprofen degradation in this system. Fig. 4c, d, and f presents the effect of the ^•^OH scavenger *tert*-butanol (0–5 mM) on sonodegradation. The rate constants for both compounds decreased progressively with increasing *tert*-butanol concentration, which can be explained by its reaction with H_2_O_2_ and/or ^•^OH, thereby competing with the target compounds for oxidizing species. Reaction pathways leading to CO_2_ and H_2_O further reduce the availability of ^•^OH for target degradation. Similar inhibitory behavior has been observed with methanol [27], [40]. These scavenger studies collectively support an ^•^OH-dependent sonodegradation mechanism for the target pharmaceuticals.

Fig. 1e and f examines the role of radical promoters (CCl_4_) and scavengers (methanol) on verapamil removal and H_2_O_2_ production to assess the contribution of free ^•^OH during US treatment [38]. In Fig. 1e, verapamil removal remained close to 100% across all CCl_4_ concentrations, whereas H_2_O_2_ production increased with increasing CCl_4_ concentration. This behavior is attributed to the sonolytic decomposition of CCl_4_: acting as an H^•^ scavenger, CCl_4_ reduces H^•^ concentration, thereby inhibiting H^•^/^•^OH recombination and increasing the steady-state concentration of free ^•^OH [30]. The enhanced ^•^OH pool concurrently elevates measured H_2_O_2_ (via secondary reactions) and directs more radicals toward target compound oxidation. Conversely, Fig. 1f shows that increasing methanol concentration (0–25 mM) progressively suppressed both verapamil removal (100%, 96.5%, 89.8%, and 78.2%) and H_2_O_2_ production. Methanol competes with verapamil for ^•^OH; because approximately 3 mM H_2_O_2_ is required to oxidize 1 mM methanol, it acts as an efficient ^•^OH scavenger, reducing radical availability for verapamil degradation. Together, these results confirm that ^•^OH is the primary oxidant in the US/Ti_3_C_2_T_x_ system [38].

Free-radical quenching tests were conducted to elucidate the dominant reactive species involved in the US/V_4_AlC_3_/PMS process for levofloxacin degradation [64]. BQ, isopropanol (IPA), and formic acid (FA) were employed as scavengers for O_2_^•−^, ^•^OH, and photogenerated h^+^, respectively. As depicted in Fig. 3b, the removal efficiency was 88.4% in the absence of any scavenger. However, upon addition of IPA, FA, and BQ, the efficiency decreased to 80.6%, 75.4%, and 42.8%, respectively. These results indicate that ^•^OH and h^+^ make relatively minor contributions, whereas O_2_^•−^ is the primary reactive species responsible for levofloxacin degradation. Furthermore, the generation of ^•^OH was verified using *o*-phenylenediamine as a probe. Its reaction with ^•^OH yields 2,3-diaminophenazine, which exhibits a characteristic absorption maximum at 435 nm [72] (Fig. 3c). The progressive increase in absorbance at 435 nm with reaction time confirms ^•^OH formation during the sonocatalytic process.

A previous study demonstrated that NH_2_-MIL-125(Ti) can act as an auxiliary material to enhance dye degradation under ultrasonic irradiation through a combination of adsorption and sonocatalytic degradation [73]. Based on its structure and composition, MIL-125(Ti)@Ti_3_C_2_T_x_ was therefore expected to exhibit similar dual-function behavior for rhodamine B degradation [74]. Indeed, rhodamine B removal under MIL-125(Ti)@Ti_3_C_2_T_x_/stirring conditions confirmed its substantial adsorption capacity. However, the noticeable differences in removal efficiencies among treatments indicate that the synergy between US irradiation and MIL-125(Ti)@Ti_3_C_2_T_x_ is predominantly due to sonocatalytic degradation. To further elucidate this mechanism, radical scavengers and promoters were introduced into the system. IPA and BQ were used to selectively quench ^•^OH and O_2_^•−^, respectively, whereas peroxydisulfate (PDS) was employed to generate additional radicals, including SO_4_^•−^ [23]. As expected, the presence of IPA and BQ markedly suppressed rhodamine B removal, leading to higher absorbance at 554 nm. The removal efficiency decreased to 75.1% in the presence of IPA and to 27.7% with BQ. In contrast, the addition of PDS further enhanced rhodamine B removal. These results indicate that reactive radical generation is the primary driver of dye degradation, with O_2_^•−^ playing a more dominant role than ^•^OH in this system. Consequently, although adsorption by MIL-125(Ti)@Ti_3_C_2_T_x_ is significant, sonocatalytic degradation constitutes the major contribution to dye removal [74].

Promoters and scavengers are powerful tools for enhancing and elucidating sonocatalytic performance and mechanisms·H_2_O_2_ and CCl_4_ enhance degradation by increasing free ^•^OH and H_2_O_2_ generation, whereas excess H_2_O_2_ or alcohols (*tert*-butanol and methanol) competitively scavenge ^•^OH, reducing PhAC degradation and confirming ^•^OH-dominated pathways in Ti_3_C_2_T_x_ systems. In contrast, quenching results with BQ, IPA, and FA in the US/V_4_AlC_3_/PMS and the MIL-125(Ti)@Ti_3_C_2_T_x_/US systems reveal O_2_^•−^ as the principal oxidant, with ^•^OH and h^+^ playing secondary roles. The addition of PDS further enhances dye degradation through the formation of additional radicals (SO_4_^•−^).

#### 2.1.5 Effects of US operation parameters

US frequency, power, and reactor type jointly influence cavitation behavior and, consequently, sonocatalytic efficacy [40]. Frequency affects bubble size, collapse intensity, and dominant ROS, while power controls cavitation number, bubble population, collapse energy, local heating, and mass transfer [75]. Reactor type and geometry (horn vs bath, plate transducers, batch vs flow), transducer placement, and volume affect energy distribution, cavitation uniformity, catalyst contact, and scalability, thereby critically influencing reaction rates and reproducibility [76].

**Fig. 5:**
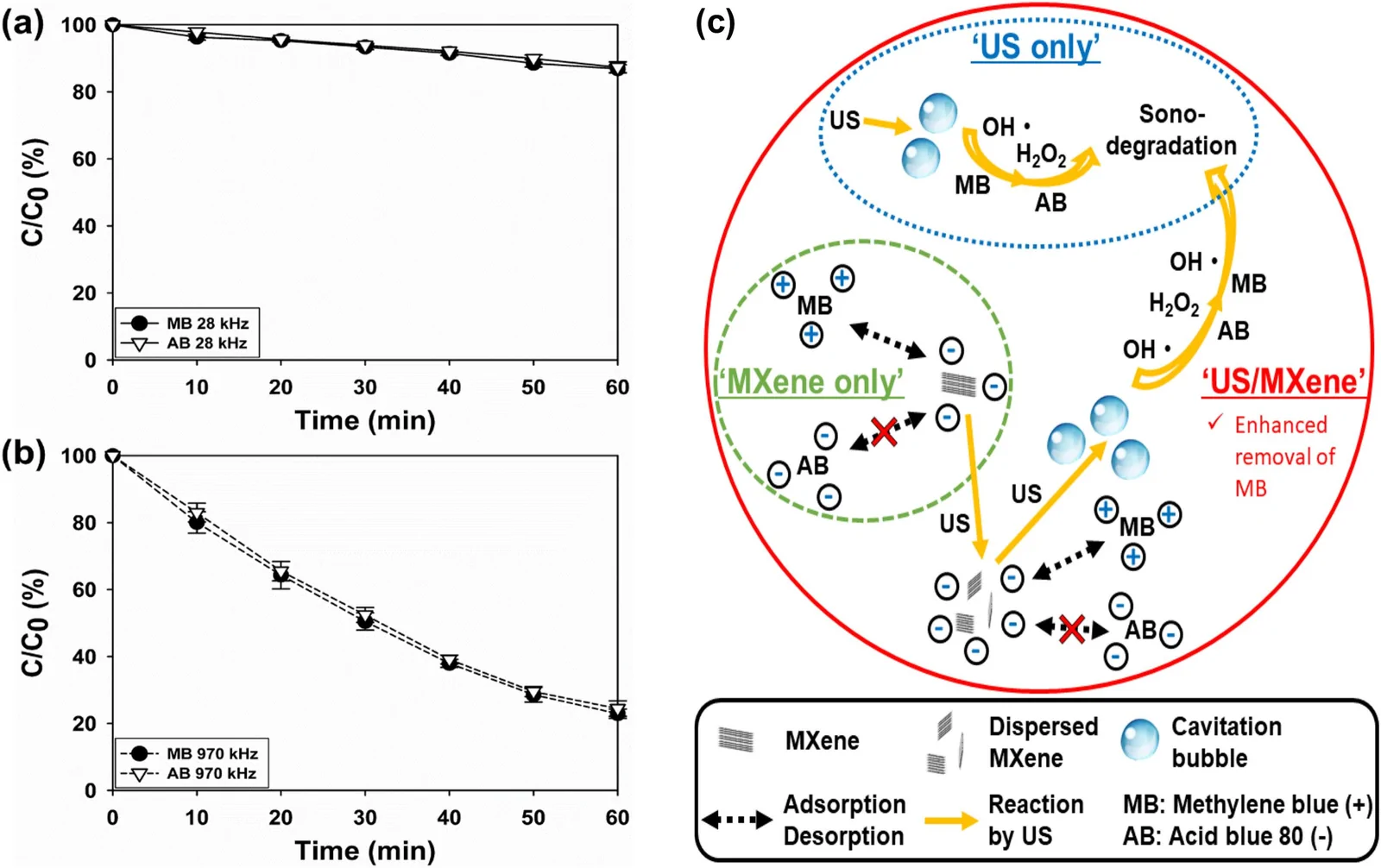
Influence of (a) 28 kHz and (b) 970 kHz US frequency on methylene blue (MB) and acid blue 80 (AB) sonocatalytic degradation (45 mg/L Ti_3_C_2_T_x_, 180 W/L, pH 7, and 293 K). (c) Proposed mechanisms related to removal of dyes in the absence or presence of MXene in US processes [57].

Between 27 and 970 kHz in the presence of Ti_3_C_2_T_x_, 27 kHz produces fewer, larger bubbles owing to energy conservation, whereas 970 kHz yields many smaller bubbles, resulting in distinct H_2_O_2_ formation trends [57]. At elevated frequencies, ^•^OH is more likely to react with dissolved organic substrates than to undergo recombination, thereby enhancing effective oxidant utilization [30]. Methylene blue and acid blue 80 degradation rates followed the measured H_2_O_2_ trends, supporting prior reports on frequency-dependent sonochemical activity [27], [42]. No substantial difference in degradation kinetics between methylene blue and acid blue 80 was observed at identical US frequencies (Fig. 5a and b). Although sonochemical oxidation is often more efficient for hydrophobic compounds due to their preferential partitioning into the high-temperature cavitation core [77], both dyes exhibit similar hydrophilic character, which likely mitigates differential partitioning and explains the comparable degradation rates [57].

Fig. 1c presents verapamil removal and H_2_O_2_ production as a function of US power in the presence of Ti_3_C_2_T_x_ [38]. Both verapamil degradation and H_2_O_2_ generation increased with US intensity, with verapamil removal efficiencies of 90.8% (90 W/L), 96.9% (135 W/L), and >99% at 180 and 225 W/L. Elevated US power promotes the formation of a greater number of cavitation bubbles, increases local temperature and pressure, and enhances the probability of effective collisions between target molecules and reactive radicals, thereby increasing oxidative degradation and H_2_O_2_ formation [78].

Ultrasonic power is a critical operating factor governing the efficiency of ciprofloxacin degradation in NiFe_2_O_4_-Bi_2_WO_6_@Ti_3_C_2_T_x_ sonocatalytic systems [66]. The apparent rate constant for ciprofloxacin degradation increased from 0.014 to 0.062 min^−1^ when the ultrasonic power was elevated from 80 to 320 W. This enhancement can be attributed to the higher generation of ^•^OH and ^•^H via H_2_O dissociation at increased power levels [42]. In addition, higher ultrasonic power promotes the formation of more active sites on the sonocatalyst surface, thereby enhancing its catalytic activity. The intensified acoustic power also strengthens microturbulence, which improves mass transfer of ciprofloxacin molecules between the bulk solution and the catalyst surface [79]. Therefore, elevated ultrasonic power is advantageous for the sonocatalytic removal of ciprofloxacin.

Ultrasound frequency also plays an important role in determining ciprofloxacin degradation under single-frequency and dual-frequency ultrasound in NiFe_2_O_4_-Bi_2_WO_6_@Ti_3_C_2_T_x_ sonocatalytic systems [66]. The sonocatalytic degradation rate followed the order 20 kHz < 40 kHz < (20 kHz + 40 kHz), indicating that dual-frequency irradiation slightly improves ciprofloxacin removal relative to single-frequency operation. However, considering that dual-frequency systems are associated with substantially higher equipment costs, the use of single-frequency ultrasound at 20 kHz is considered more practical for ciprofloxacin treatment despite the modest performance gain afforded by frequency modulation.

Ultrasonic operating conditions critically affect MXene-based sonocatalysis by dictating cavitation intensity, ROS production, and mass transfer. Frequency determines bubble size and ^•^OH utilization, with higher or dual frequencies generating more numerous, smaller bubbles and enhanced H_2_O_2_-correlated degradation, as demonstrated for dyes and PhACs. Ultrasonic power strongly promotes organic contaminant removal by increasing bubble population, collapse energy, active-site formation, and microturbulence. Reactor configuration and energy distribution (horn vs bath, geometry, and volume) further modulate cavitation uniformity and scalability. Optimized combinations of frequency, power, and reactor configuration are essential for maximizing ROS-driven degradation while maintaining process practicality.

### 2.2 Degradation affected by hybrid processes involving UV or Fenton

UV and Fenton processes synergize with sonocatalysis by boosting ROS production and enabling complementary pathways: (i) UV (photons) activates photocatalysts and generates e^−^/h^+^ and H_2_O_2_/^•^OH, improving charge separation when combined with ultrasound [80], (ii) Fenton (Fe^2+^/H_2_O_2_) under ultrasound accelerates Fe^2+^/Fe^3+^ cycling, increases ^•^OH generation, and reduces sludge formation [81], and (iii) hybrid systems can enhance degradation rates and mineralization, but require careful control of scavenging effects, catalyst/iron leaching, byproduct formation, and energy/cost trade-offs [23].

To evaluate the influence of the piezoelectric effect of Bi_2_WO_6_-Ag@Ti_3_C_2_T_x_ on sono-photocatalytic performance, rhodamine B degradation was measured under visible light (λ > 380 nm and 100 mW/cm^2^), US (100 W and 40 kHz), and combined excitation [82]. Under sole light or US irradiation, pristine Bi_2_WO_6_ achieved 33% and 17% degradation in 60 min, respectively, indicating slow charge transfer and pronounced bulk recombination. However, Bi_2_WO_6_-Ag@Ti_3_C_2_T_x_ exhibited improved degradation under identical conditions, attributable to Ag-induced localized surface plasmon resonance. In particular, co-excitation (light + US) enhanced Bi_2_WO_6_-Ag@Ti_3_C_2_T_x_ degradation to 77.3% in 20 min, with a first-order rate constant (*k*) of 0.072 min^−1^ approximately 3.3 times higher than that of pristine Bi_2_WO_6_. This pronounced synergistic effect is attributed to piezoelectric polarization: mechanically induced piezoelectric charge reduces the interfacial Schottky barrier, accelerates charge migration, and suppresses recombination [83]. The catalyst retained >86% removal after five cycles and preserved 87% of its initial *k* value, demonstrating stable performance and practical potential [82].

ZnO/CdSe/Ti_3_C_2_ MXene sonophotocatalysts were prepared via a simple and reliable method involving calcination at 400 °C [84]. The resulting nanocomposites exhibited excellent sonophotocatalytic performance, achieving removal rates of 99.99%, 99.98%, and 99.90% for ciprofloxacin, amoxicillin, and ofloxacin, respectively, under combined US irradiation and visible light. In this system, US promotes the generation of reactive species and enhances mass transfer, while photocatalysis utilizes light energy to activate the catalyst and drive pollutant degradation. A comparative study was conducted under three different conditions to evaluate the role of the sonocatalyst: (i) only contaminants and visible light were present without catalyst; (ii) contaminants and US alone in the absence of visible light and catalyst; and (iii) contaminants, US, and catalyst without visible light. These findings indicate that US alone can partially degrade antibiotics, while its efficiency is significantly enhanced in the presence of the catalyst. US-induced cavitation generates microbubbles that rapidly collapse, producing localized high temperature and pressure and consequently yielding highly reactive species such as ^•^OH [36]. In addition, ultrasonic waves impart mechanical effects that can weaken or disrupt chemical bonds in contaminant molecules. The physical agitation of the medium improves mass transfer, increasing contact between contaminants and reactive species and thereby accelerating degradation [26]. Furthermore, acoustic streaming, caused by ultrasonic vibrations, induces fluid flow near surfaces and boundaries, enhancing the transport and distribution of reactants and products and facilitating the breakdown of contaminants [85].

The tetracycline degradation rate was evaluated to elucidate the sonophotocatalytic performance of the TiO_2_-WS_2_@Ti_3_C_2_T_x_ hybrid heterostructure [86]. Under combined US and visible-light irradiation, TiO_2_-WS_2_@Ti_3_C_2_T_x_ achieved 98.9% tetracycline degradation within 90 min, markedly surpassing adsorption (14.1%), sonolysis (18.1%), sonocatalysis (57.1%), and photocatalysis (89.9%). The enhanced performance is attributed to the synergistic contribution of visible-light excitation and ultrasonic cavitation [87]. In the absence of light, TiO_2_-WS_2_@Ti_3_C_2_T_x_ showed a tetracycline degradation efficiency of 14.2%, primarily owing to static interactions between surface charges and tetracycline, as well as defect states capable of adsorbing pollutant ions [88]. Under light-driven sonophotocatalytic conditions, more efficient separation of photogenerated charge carriers and the concomitant formation of ROS facilitated tetracycline degradation and ultimately led to mineralization of organic pollutants into CO_2_ and H_2_O. Owing to its strong photo response, the TiO_2_-WS_2_@Ti_3_C_2_T_x_ heterostructure displayed superior photocatalytic activity toward tetracycline compared with other heterostructure configurations examined. After 60 min of irradiation, TiO_2_-WS_2_@Ti_3_C_2_T_x_ exhibited a removal efficiency approximately 2.5, 2.0, 1.7, and 1.2 times higher than those of Ti_3_C_2_T_x_, WS_2_, TiO_2_/Ti_3_C_2_T_x_, and WS_2_-Ti_3_C_2_T_x_, respectively. The corresponding sonophotocatalytic degradation efficiencies of Ti_3_C_2_T_x_, WS_2_, TiO_2_/Ti_3_C_2_T_x_, WS_2_-Ti_3_C_2_T_x_, and the system without catalyst were 36.2%, 45.3%, 54.1%, 72.1%, and 13.1%, respectively [86]. The improved performance upon incorporation of WS_2_ nanoflakes onto the Ti_3_C_2_T_x_ surface confirms the beneficial effect of constructing the ternary heterostructure. The presence of TiO_2_ nanograins at the WS_2_-Ti_3_C_2_T_x_ interface effectively modulates the relative conduction band positions of Ti_3_C_2_T_x_ and WS_2_, thereby enhancing interfacial charge separation [86].

The relatively low efficiency of Fe(II) regeneration remains a key limitation of conventional Fenton systems [89]. To overcome this limitation, novel SmFeO_3_/Ti_3_C_2_T_x_ catalysts were prepared via a self-assembly technique and applied to the sono-Fenton degradation of tetracycline [90]. To elucidate the degradation mechanism, radical scavenging experiments were conducted using *tert*-butanol, BQ, and NaN_3_ as quenchers for ^•^OH, O_2_^•−^, and ^1^O_2_, respectively. Upon the addition of these scavengers, the degradation efficiency of tetracycline decreased to 42%, 78%, and 84%, respectively, suggesting that ^•^OH is the predominant reactive species in this system [91]. The combined use of ultrasound and Fenton chemistry for tetracycline degradation can still be limited under certain conditions [92]. The sono-Fenton process resembles the traditional Fenton reaction, while the SmFeO_3_/Ti_3_C_2_T_x_ composite enhances pollutant removal due to the formation of a Schottky junction [93]. In heterogeneous Fenton systems, ultrasound promotes the reduction of Fe^3+^ to Fe^2+^ and facilitates H_2_O_2_ decomposition on the SmFeO_3_/Ti_3_C_2_T_x_ surface, generating ^•^OH and HO_2_^•^ [94]. Ultrasonic cavitation produces transient light emission and localized “hot spots”; the resulting sonoluminescence can excite the SmFeO_3_/Ti_3_C_2_T_x_ catalyst to form e^−^/h^+^ pairs on SmFeO_3_ nanoparticles [95], while the high-temperature microenvironments accelerate H_2_O_2_ pyrolysis to additional ^•^OH on the catalyst surface [96]. Accordingly, tetracycline degradation over SmFeO_3_/Ti_3_C_2_T_x_ under sono-Fenton conditions is proposed to proceed via two synergistic pathways: (i) a heterogeneous (conventional) Fenton-like pathway and (ii) a sonocatalytic pathway, in which SmFeO_3_/Ti_3_C_2_T_x_ absorbs ultrasonic energy, leading to charge separation. The Schottky junction efficiently drives e^−^ transfer from SmFeO_3_ to Ti_3_C_2_T_x_, and the e^−^ captured by Ti_3_C_2_T_x_ are prevented from recombining with h^+^ in the SmFeO_3_ conduction band, thereby suppressing charge recombination in the composite [97]. These accumulated e^−^ on the Ti_3_C_2_T_x_ surface subsequently react with H_2_O_2_ and dissolved O_2_ to form ^•^OH and O_2_^•−^. Ultimately, the generated reactive species mineralize tetracycline to CO_2_ and H_2_O [90].

Hybrid UV/visible-US and sono-Fenton processes integrated with MXenes substantially enhance antibiotic and dye removal by coupling cavitation with photochemical and Fenton pathways. In Bi_2_WO_6_-Ag@Ti_3_C_2_T_x_, piezoelectric polarization and plasmonic Ag under co-excitation greatly accelerate charge separation and rhodamine B degradation. ZnO/CdSe/Ti_3_C_2_ and TiO_2_-WS_2_@Ti_3_C_2_T_x_ achieve near-complete sonophotocatalytic antibiotic and tetracycline removal via combined ROS generation and interfacial heterostructures. SmFeO_3_/Ti_3_C_2_T_x_ enables efficient sono-Fenton tetracycline degradation through Schottky-driven Fe^2+^/Fe^3+^ cycling, enhanced ^•^OH/O_2_^•−^ production, and sonoluminescence-assisted e^−^/h^+^ generation. Synergistic light-US-Fenton systems substantially boost ROS yield, charge utilization, and mineralization efficiency.

### 2.3 Degradation mechanisms and mineralization pathways

The significance of determining the degradation mechanism in MXene-based sonocatalytic systems lies in elucidating the precise pathways of contaminant breakdown, including ^•^OH generation and surface interactions under ultrasonic irradiation [98]. This understanding can support the optimization of catalyst design, enhance reaction efficiency, and minimize the formation of unintended byproducts, thereby ensuring safer and more sustainable water and wastewater treatment processes [99].

A fluoropolymer-based porous membrane incorporating nanocrystal cellulose and Ti_3_C_2_T_x_ (NC-Ti_3_C_2_T_x_) within a polyvinylidene fluoride (PVDF) matrix was prepared, achieving a relatively high structural porosity uniformity (59.3%) compared with the pristine PVDF membrane [100]. The degradation mechanism of rhodamine B in the presence of the NC-Ti_3_C_2_T_x_ membrane under sonocatalytic conditions was investigated via radical-trapping experiments. Scavenger tests demonstrated that the addition of ethylenediaminetetraacetic acid (EDTA; h^+^ scavenger) resulted in only a slight decrease in rhodamine B removal, indicating that h^+^ species play a minor role [101]. In contrast, isopropanol (^•^OH scavenger) caused pronounced inhibition of degradation, demonstrating that ^•^OH radicals play a dominant role in rhodamine B oxidation. The addition of benzoquinone (O_2_^•−^ scavenger) also significantly reduced degradation efficiency, indicating that O_2_^•−^ as a secondary reactive species. These results suggest that sono-generated reactive oxygen species (primarily ^•^OH, with contributory O_2_^•−^) govern rhodamine B degradation on the NC-Ti_3_C_2_T_x_ membrane, supporting its dual functionality for water filtration and effective dye remediation [100].

The synergistic indices of Ti_3_C_2_T_x_ in the Ti_3_C_2_T_x_/US hybrid process were evaluated during the degradation of methylene blue and acid blue 80 to assess the feasibility of Ti_3_C_2_T_x_ as a sonocatalyst in US degradation processes [57], [102]. Apparent first-order rate constants were used to calculate synergistic indices as follows: [*k*_1_(US/MXene)/*k*_1_(US only) + *k*_1_(MXene only)]. The synergistic indices for methylene blue ranged from 1.21 to 1.86, whereas acid blue 80 exhibited lower values (1.00–1.11), indicating synergistic enhancement of the combined MXene/US process for both dyes, with a substantially greater improvement for methylene blue. Two mechanisms are proposed to account for this behavior: (i) MXene particles act as additional cavitation nuclei under US, promoting cavitation collapse and increasing ^•^OH generation from water [103]; and (ii) US facilitates exposure of MXene active sites and reduces particle size, thereby enhancing adsorption of dye molecules [36]. Dynamic light scattering analysis supports mechanism (ii), showing a reduction in the average MXene hydrodynamic diameter from 610 nm to 377 nm after 28 kHz US treatment [57]. The substantially lower synergistic index for acid blue 80 is attributed to electrostatic effects, as both MXene and acid blue 80 are negatively charged, minimizing adsorption-driven enhancement and effectively limiting mechanism (ii) for acid blue 80 [38]. A schematic of the proposed removal pathways, with and without MXene under ultrasound, is presented in Fig. 5c.

**Fig. 6:**
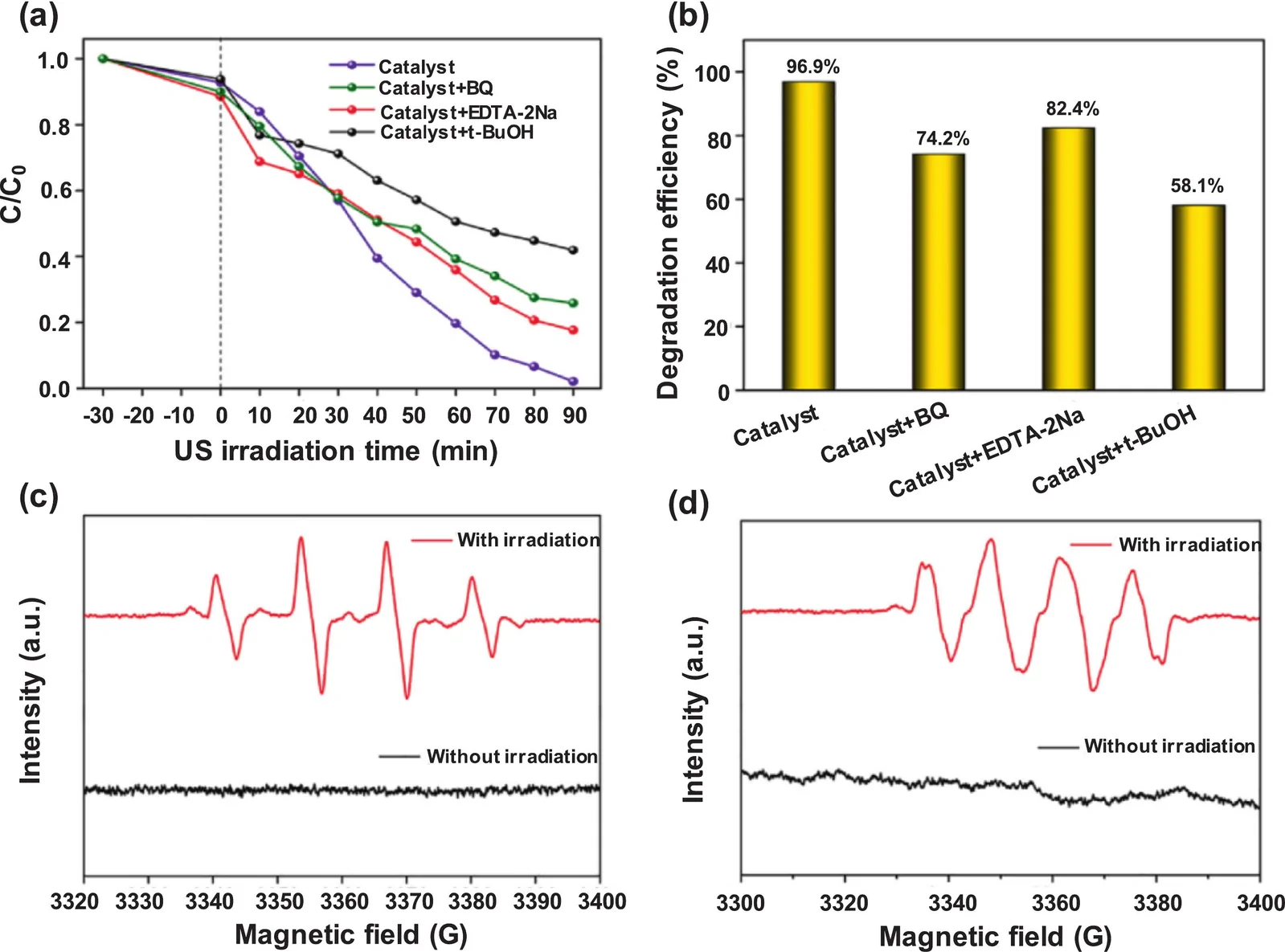
(a) Effects of different scavengers on the degradation of bisphenol A over CoTiO_3_@Ti_3_C_2_T_x_ (1:0.5) nanocomposite and (b) the corresponding degradation removal efficiency. (c) DMPO-^•^OH spin trapping ESR spectra of CoTiO_3_@Ti_3_C_2_T_x_ (1:0.5) nanocomposite in an aqueous dispersion and (d) DMPO-O_2_^•−^ spectra CoTiO_3_@Ti_3_C_2_T_x_ (1:0.5) in methanol dispersion [44].

To elucidate the reactive oxygen species responsible for bisphenol A degradation by the CoTiO_3_@Ti_3_C_2_T_x_ catalyst, radical scavenging experiments and electron spin resonance (ESR) measurements were conducted (Fig. 6a and b) [44]. *tert*-Butanol (a well-known ^•^OH scavenger; *k* = 3.8–7.6 × 10^8^ M^−1^ s^−1^) was first introduced into the US/CoTiO_3_@Ti_3_C_2_T_x_ (1:0.5) system, resulting in a 38.8% decrease in bisphenol A degradation, indicating a major role of ^•^OH in the sonocatalytic process. Nevertheless, 58.1% removal remained, suggesting the participation of additional ROSs. The addition of benzoquinone reduced degradation efficiency to 74.2%, confirming the involvement of O_2_^•−^. To probe the contribution of photogenerated h^+^, disodium ethylenediaminetetraacetic acid was introduced, resulting in only a slight decrease in bisphenol A removal, indicating a minor role for valence-band h^+^ in the degradation pathway. ESR spectroscopy further validated these findings. Under ultrasonic irradiation of CoTiO_3_@Ti_3_C_2_T_x_, the 5,5-dimethyl-1-pyrroline N-oxide (DMPO) spin trap produced a four-line pattern with a 1:2:2:1 intensity ratio, characteristic of the DMPO-^•^OH adduct (Fig. 6c), while a distinct six-line spectrum corresponding to the DMPO-O_2_^•−^ adduct was also observed (Fig. 6d). No ESR signals were detected in the absence of ultrasound, confirming that ROS generation arises from sonication (cavitation and sonoluminescence) in the CoTiO_3_@Ti_3_C_2_T_x_ system [23]. These results demonstrate that ^•^OH and O_2_^•−^ are the primary ROSs responsible for bisphenol A degradation, with a secondary contribution from photogenerated h^+^ [44].

Mineralization pathways describe how contaminants are sequentially oxidized to CO_2_, H_2_O and inorganic ions. These pathways (e.g., hydroxylation, dealkylation, dechlorination, ring opening, and cleavage into small organic acids) determine intermediate formation, reaction kinetics, and residual toxicity [103]. While dominant ROSs and catalyst surface reactions govern these pathways, water quality parameters (e.g., pH, temperature, and background ions) influence both reaction routes and rates [75]. Understanding these pathways is therefore essential for process optimization, identification of recalcitrant intermediates or byproduct risks, and the design of monitoring strategies [e.g., total organic carbon (TOC), inorganic ions, and toxicity assays] to achieve complete mineralization [104].

Based on experimental data and theoretical simulations (finite element method; COMSOL Multiphysics), a coherent piezo-sono/photocatalytic mechanism was proposed for rhodamine B degradation in the presence of Bi_2_WO_6_-Ag@Ti_3_C_2_T_x_ [82]. Ag nanoparticles and Ti_3_C_2_T_x_ form dual Schottky junctions with Bi_2_WO_6_, reshaping its energy-band structure and promoting unidirectional charge transfer [105]. Under visible-light irradiation, plasmonic excitation of Ag generates hot e^−^ which are injected into the conduction band of Bi_2_WO_6_ and subsequently transferred to Ti_3_C_2_T_x_. Simultaneously ultrasonic excitation induces piezoelectric polarization in Ti_3_C_2_T_x_, lowering interfacial Schottky barriers, enhancing charge separation, and accelerating carrier migration [33]. Consequently, the combined plasmonic and piezoelectric effects promote electron accumulation on Ti_3_C_2_T_x_ and facilitate O_2_ activation to generate O_2_^•−^. Liquid chromatography–mass spectrometry analysis supports the proposed degradation pathway: the generated O_2_^•−^ species preferentially attack the N site of rhodamine B, initiating N-demethylation and/or hydroxylation of the C–N^+^ moiety [106]. Subsequent demethylation and hydroxylation steps lead to aromatic ring opening and progressive fragmentation into smaller, more oxidized intermediates. The enhanced mineralization of rhodamine B is attributed to synergistic plasmonic hot e^−^ injection, piezoelectric-field-induced reduction of Schottky barriers, and Ti_3_C_2_T_x_-facilitated oxygen activation [82].

TOC analyses were conducted to evaluate acetaminophen mineralization using Ti_3_C_2_T_x_-MIL [41]. Sonolysis alone achieved approximately 7% mineralization, consistent with its limited degradation efficiency. In contrast, sonocatalytic treatment with Ti_3_C_2_T_x_-MIL achieved significantly higher mineralization (approximately 72%). These findings support a mechanism in which sonoluminescence provides photon energy that excites the Ti_3_C_2_T_X_-MIL composite, while cavitation-induced hot spots generate e^−^/h^+^ pairs at the catalyst surface [107]. Mott–Schottky analysis yielded flat-band potentials (E_fb_ vs. Ag/AgCl) of −0.29 V for MXene and −0.76 V for MOF, corresponding to −0.09 V and −0.56 V versus the normal hydrogen electrode after Nernst conversion. The positive slopes of the Mott–Schottky plots indicate n-type semiconductor behavior, consistent with previous reports [108]. Under US irradiation, electrons in the valence band of the MOF are excited to the conduction band, leaving holes in the valence band. These photoexcited e^−^ are subsequently transferred to Ti_3_C_2_T_x_, facilitated by its partially oxidized surface, which enhances e^−^ transport and suppresses e^−^/h^+^ recombination. The accumulated electrons on Ti_3_C_2_T_x_ reduce H_2_O_2_ and dissolved O_2_ to generate ^•^OH and O_2_^•−^, while h^+^ on the MOF oxidize water to produce ^•^OH [41]. These reactive oxygen species subsequently mineralize pharmaceutical contaminants adsorbed on the catalyst surface, as illustrated in Fig. 2b.

Mechanistic elucidation in MXene-based sonocatalysis is essential for rational catalyst design, process optimization, and safe water treatment. Ti-based, partially oxidized MXenes with conductive surfaces and abundant –O/–OH groups favor interfacial electron transfer, H_2_O_2_ activation, and ^•^OH generation. Electron-rich interfaces and oxygen-accessible terminations promote O_2_ reduction and O_2_^•−^ production. –F-rich surfaces primarily influence hydrophilicity, contaminant adsorption, and cavitation proximity, thereby indirectly affecting ROS exposure. V-, Nb-, and Mo-based MXenes, as well as MXene heterostructures, can alter redox potentials and redirect charge-transfer pathways, potentially changing the predominant ROS.

## 3 Biocompatibility and toxicity of MXene-based sonocatalysts

Biocompatibility is a critical prerequisite for translating nanostructured materials into practical biomedical applications [109]. Therefore, the cytotoxicity of MoO_x_@Mo_2_C nanonetworks was first evaluated using mesenchymal stem cells (MSCs) and human umbilical vein endothelial cells [110]. The results indicated that MoO_x_@Mo_2_C nanonetworks exhibited favorable cytocompatibility, with no apparent adverse effects on cell viability. Cellular responses were also assessed after several treatments, including ultrasound stimulation. MSC viability and morphology were examined using live/dead fluorescence staining and confocal microscopy. The predominance of strong green fluorescence, together with well-maintained cell morphology, confirmed that the sonodynamic activity induced by MoO_x_@Mo_2_C nanonetworks had no detectable damage to normal cells. In addition, confocal images showed no obvious aggregation between normal cells and the nanonetworks, suggesting that MoO_x_@Mo_2_C nanonetworks exhibited insignificant nonspecific capture behavior toward healthy cells. The biodegradability of polyvinyl pyrrolidone (PVP)-modified MoO_x_@Mo_2_C nanonetworks was further investigated at different pH values. Digital images and UV–vis absorbance spectra showed that MoO_x_@Mo_2_C@PVP nanonetworks maintained their structural integrity under mildly acidic conditions (pH 5.4), whereas gradual degradation occurred under physiological and mildly alkaline conditions (pH 7.4 and 9.4) within 72 h. This pH-responsive degradation behavior suggests that the material can remain stable in acidic infection microenvironments to exert antibacterial activity, while undergoing self-degradation in normal tissues after treatment [111]. For systemic biosafety evaluation, mice were intravenously injected with MoO_x_@Mo_2_C nanonetworks or saline controls. PVP modification improved colloidal stability and syringeability without altering the network-like morphology or antibacterial performance. Hematological and biochemical indicators showed no significant differences from controls, indicating negligible hematological, hepatic, or renal toxicity [110].

The potential ecological risks associated with levofloxacin and its transformation products generated during the US/V_4_AlC_3_ MAX phase/PMS process were assessed using the Ecological Structure–Activity Relationships (ECOSAR) model [64]. Acute and chronic toxicities were predicted for representative aquatic organisms, including fish, green algae, and daphnia, based on the half-lethal (LC_50_), half-effective concentration (EC_50_), and chronic toxicity (ChV) values. The predicted toxicity levels (LC_50_/EC_50_/ChV) were categorized as extremely toxic (<1 mg/L), toxic (1–10 mg/L), harmful (10–100 mg/L), and not harmful (>100 mg/L). Most degradation intermediates exhibited substantially reduced acute toxicity compared with the parent levofloxacin molecule. The final transformation products were predicted to fall within the non-toxic range for acute toxicity toward fish, green algae, and daphnia. Similarly, the chronic toxicity assessment indicated that the majority of end products were environmentally benign, which showed potential chronic toxicity in the daphnia model. The ECOSAR results suggest that the proposed degradation routes generally reduce the ecological hazard of levofloxacin during treatment, indicating that the US/V_4_AlC_3_ MAX phase/PMS system can effectively degrade levofloxacin while minimizing the formation of persistently toxic by-products [64].

## 4 Stability and reusability of MXene-based sonocatalysts

To ensure long-term viability and prevent activity loss, sonocatalysts must exhibit both stability and reusability, achieved by maintaining their structural integrity, surface area, and active sites under ultrasonic stress [112]. Low metal leaching and resistance to fouling or sintering are essential for preserving catalytic performance and preventing secondary contamination. Adequate reusability was demonstrated through facile regeneration (e.g., washing, thermal, or chemical treatment) and sustained activity over multiple cycles [101]. Performance evaluations, including recycling tests and leaching analyses, provide important insights for process optimization and scale-up.

For NC-Ti_3_C_2_T_x_, membrane stability and sonocatalytic reusability were evaluated over five consecutive cycles, with rinsing between runs [100]. The rhodamine B degradation efficiency gradually decreased from 97.6% in the initial cycle to 83.1% in the fifth cycle. Post-use characterization revealed no observable loss of structural integrity in the NC-Ti_3_C_2_T_x_ composite membrane, indicating good durability. The sustained performance is attributed to the robust membrane architecture, which prevents catalyst aggregation within the polymer matrix and preserves active surface sites during repeated sonocatalytic operation [100].

Methylene blue-adsorbed Ti_3_C_2_T_x_ MXene was regenerated using 1 M HCl over five cycles [57]. Reusability was evaluated through X-ray diffraction (XRD) crystallinity analysis and normalized methylene blue removal efficiency. The XRD pattern of pristine MXene was consistent with literature reports [113], [114], and crystallinity was quantified by summing the intensities of the ten most prominent XRD peaks for each sample. This approach enables assessment of structural deterioration during repeated adsorption-sonication cycles [115]. The MXene retained high crystallinity after five regeneration cycles at both ultrasonic frequencies (28 and 970 kHz), indicating strong structural stability under operational conditions. Correspondingly, normalized methylene blue removal remained above 90% in both frequency regimes after five cycles. These results demonstrate that MXene exhibits robust structural and functional stability during repeated sonocatalytic regeneration, supporting its practical application in dye-containing wastewater treatment [57].

Reusability is a critical factor for the large-scale application of catalysts. To assess the sonocatalytic performance of the regenerated V_4_AlC_3_ catalyst, five consecutive reuse cycles were conducted under identical conditions [64]. As shown in Fig. 3d, the levofloxacin removal efficiency decreased by only 10.4% after five cycles, indicating excellent reusability of the synthesized catalyst. Structural stability was further evaluated using scanning electron microscopy (SEM) and XRD analyses. The morphology of V_4_AlC_3_ remained essentially unchanged after five cycles, and no significant differences were observed in the XRD patterns before and after cycling. In addition, inductively coupled plasma optical emission spectroscopy was employed to assess metal leaching of V and Al after each cycle. The maximum concentrations of V and Al were 0.147 and 0.188 mg/L, respectively, both below the limits specified in the national discharge standards [116], [117]. These findings collectively confirm the high reusability and structural stability of the V_4_AlC_3_ sonocatalyst [64].

The stability of the ZnO/CdSe/Ti_3_C_2_ nanocomposite was also evaluated using Fourier transform infrared (FTIR) spectroscopy following sonophotocatalytic degradation of ciprofloxacin, amoxicillin, and ofloxacin [84]. After each run, the catalyst powder was collected, washed several times with deionized water, and dried at 70 °C for 24 h. The recovered ZnO/CdSe/Ti_3_C_2_ was subsequently reused for four consecutive cycles. The photodegradation efficiency (greater than 99%) remained essentially unchanged compared with the initial run, indicating excellent recyclability. The photocurrent response of ZnO/CdSe/Ti_3_C_2_ after four cycles further confirmed that its photoelectrochemical performance was well preserved during repeated use. Moreover, FTIR spectra obtained before and after cycling showed no significant changes in characteristic bands, demonstrating that the structural integrity and chemical stability of the catalyst were maintained after repeated sonophotocatalytic operation [84].

The sonocatalytic ciprofloxacin degradation efficiency of NiFe_2_O_4_-Bi_2_WO_6_@Ti_3_C_2_T_x_ decreases only marginally (less than 5%) after five consecutive cycles, indicating excellent recyclability and high operational stability [66]. This stability is further supported by FTIR and X-ray photoelectron spectroscopy (XPS) analyses, which showed negligible differences between fresh and used catalysts, indicating that the surface chemical composition remained essentially unchanged upon reuse. Additionally, XRD patterns and SEM images confirmed that the crystal structure and morphology of the NiFe_2_O_4_-Bi_2_WO_6_@Ti_3_C_2_T_x_ composite were well preserved after cycling, with no significant structural degradation. To further evaluate catalyst stability, metal ion leaching experiments were conducted. The concentrations of Fe^3+^, Ni^2+^, W^2+^, and Bi^3+^ released into solution were 4.59, 9.19, 4.97, and 17.4 μg/L, respectively, and no detectable leaching of Ti occurred during the sonocatalytic degradation process. These results demonstrate that the NiFe_2_O_4_-Bi_2_WO_6_@Ti_3_C_2_T_x_ composite possesses excellent structural and chemical stability and can be utilized over extended operational periods without significant loss of catalytic performance [66].

Long-term stability and reusability are essential for the practical application of MXene-based sonocatalysts. NC-Ti_3_C_2_T_x_ membranes retain structural integrity and more than 80% rhodamine B removal after five cycles, while Ti_3_C_2_T_x_ powders preserve crystallinity and more than 90% methylene blue removal following repeated acid regeneration and sonication. Layered V_4_AlC_3_ and ZnO/CdSe/Ti_3_C_2_ showed negligible structural changes, high antibiotic degradation efficiencies (greater than 99%), and minimal metal leaching over multiple cycles. Similarly, NiFe_2_O_4_-Bi_2_WO_6_@Ti_3_C_2_T_x_ shows less than 5% activity loss, stable crystal and surface chemistry, and ultra-low ion release. These findings collectively demonstrate strong mechanical, chemical, and catalytic durability, supporting the potential of MXene-based systems for scalable water and wastewater treatment applications. Table 1 summarizes selected MXene-based catalysts used for sonocatalytic degradation. In addition, Table S1 summarizes the removal of selected EDCs and PhACs using US treatment in the presence of established sonocatalysts for comparative evaluation.

**Table 1:** Brief summary of selected MXene-based catalysts used for sonocatalytic degradation.

Compound	Catalysts or hybrids	Experimental conditions	Key removal or rate constant	Key finding	Ref.
Rhodamine B	Nanocrystal cellulose (NC)-Ti_3_C_2_T_x_	C_0_ = 10 mg/L SDW 50 kHz; 250 W 90 min	97.6%	Demonstrated dual water-treatment functionality by combining membrane filtration, PVDF-related piezoelectric response, dye adsorption, and Ti_3_C_2_T_x_-driven sonocatalytic ROS generation.	[100]
Amitriptyline ibuprofen	Ti_3_C_2_T_x_	C_0_ = 10 mM SDW 35 kHz; 50 W/L 120 min	0.0277 min^−1^ (amitriptyline) 0.0156 min^−1^ (ibuprofen)	Humic acid may slightly enhance degradation by suppressing hydroxyl-radical recombination, while H_2_O_2_ and *tert*-butanol tests confirmed ^•^OH-mediated oxidation.	[54]
Rhodamine B	Sandwich-like Bi_2_WO_6_@Ag/Ti_3_C_2_T_x_ nanoflower spheres	SDW 40 kHz; 50 W l > 380 nm 60 min	77.3% (0.072 min^−1^) within 20 min	Established that Ag plasmonic excitation and Ti_3_C_2_T_x_-assisted piezoelectric polarization lower interfacial resistance and enhance charge separation under combined light/ultrasound.	[82]
Methylene blue acid blue 80	Ti_3_C_2_T_x_	C_0_ = 10 mg/L SDW 28/970 kHz; 45–180 W/L 60 min	0.0191 min^−1^ (970 kHz, methylene blue) 0.0135 min^−1^ (970 kHz, acid blue 80)	Demonstrated frequency-dependent degradation governed by H_2_O_2_ formation and pollutant-MXene electrostatic interactions.	[57]
Diclofenac verapamil	Single-/multi-layered Ti_3_C_2_T_x_	C_0_ = 10 mM SDW 28/570 kHz; 90–225 W/L 60 min	0.080 min^−1^ (diclofenac) 0.101 min^−1^ (verapamil); 570 kHz, single-layered Ti_3_C_2_T_x_	While the dominant mechanism was oxidation, Ti_3_C_2_T_x_ also functioned as an adsorbent. Notably, the negatively charged MXene adsorbed a greater amount of positively charged compound.	[38]
Tetracycline	SmFeO_3_/Ti_3_C_2_T_x_	C_0_ = 10 mg/L SDW 970 kHz 30 min	99% (catalyst = 500 mg/L catalyst and H_2_O_2_ = 2 mM)	Ti_3_C_2_T_x_ functions as an electron trap in a Schottky-type sono-Fenton system, accelerating carrier separation and tetracycline oxidation.	[90]
Ciprofloxacin amoxicillin ofloxacin	ZnO-CdSe@Ti_3_C_2_	C_0_ = 10 mg/L SDW 15, 30, 75 W 240 min	99.99% (ciprofloxacin) 99.98% (amoxicillin) 99.90% (ofloxacin)	The scavenger tests showed the catalyst could degrade the compounds, suggesting that ^•^O_2_^−^ play a significant role in the sonophotocatalytic degradation process.	[84]
Sulfadiazine acetaminophen	MIL-101(Cr)/Ti_3_C_2_T_x_	C_0_ = 10 mM SDW 40 kHz; 180 W/L 60 min	91.5% (sulfadiazine) 90.6% (acetaminophen)	The superior sonocatalytic performance of MIL-101(Cr)/Ti_3_C_2_T_x_ is ascribed to its optimized contact interface and the highly efficient electron-hole pair separation facilitated by the type-II heterostructure.	[41]
Tetracycline	TiO_2_/WS_2_-Ti_3_C_2_T_x_	C_0_ = 10 mg/L SDW neutral pH 120 min	0.0019, 0.0011, 0.0078, 0.0171, and 0.0341 min^−1^ (sonolysis, adsorption, sonocatalysis, photocatalysis, sonophotocatalysis)	The defect-enriched TiO_2_ interfacial layer on MXene modified the electronic structure to form a Schottky junction.	[86]
Bisphenol A	CoTiO_3_@Ti_3_C_2_T_x_	C_0_ = 10 mg/L SDW 970 kHz; 0–600 W 90 min	96.9%	The MXene nanosheets introduce new surface-active sites that enhance interactions between the catalyst and bisphenol A molecules.	[44]
Levofloxacin rhodamine B	TiO_2_@V_2_CT_x_	C_0_ = 10 mg/L SDW 970 kHz; 0–600 W 90 min	91.5% (sulfadiazine) 90.6% (acetaminophen)	V_2_CT_x_, an underexplored vanadium-based MXene, functions as both a conductive and catalytically active component.	[118]
Levofloxacin	V_4_AlC_3_ MAX phase	C_0_ = 10 mg/L SDW 37 kHz; 150 W 60 min	88.4% (distilled water)76.8% (tap water)60.2% (lake water)	The MAX phase displayed significant stability, retaining high degradation efficiency with a marginal decline of 10.4% after five consecutive uses.	[64]
Ciprofloxacin	NiFe_2_O_4_/Bi_2_WO_6_@Ti_3_C_2_	C_0_ = 10 mg/L SDW 20/40 kHz; 80–320 W 60 min	0.05176, 0.00877, and 0.00575 min^−1^ (NiFe_2_O_4_/Bi_2_WO_6_@Ti_3_C_2_, US/MXene, and US alone, respectively)	MXene bridges the Z-scheme heterojunction as an electron mediator, improving ciprofloxacin degradation and maintaining low metal leaching after reuse.	[66]
Rhodamine B micro-polyethylene	MIL-125(Ti)@Ti_3_C_2_T_x_	C_0_ = 5 mg/L SDW 35 kHz; 150 W 60 min	90% (rhodamine B)75% (micro-polyethylene)	The as-synthesized nanocomposite displayed a plicated surface morphology and porous architecture with high photosensitivity and e^-^ transfer efficiency.	[74]

C_o_ = contaminant initial concentration; SDW = synthetic drinking water; CNF = cellulose nanofiber; PVDF = β polymorphs-polyvinylidene fluoride; NOM = natural organic matter.

## 5 Perspectives and areas of future study

Future research on MXene-based sonocatalysis should move beyond proof-of-concept contaminant degradation toward mechanistically informed and scalable water treatment technologies. Although recent studies have identified dominant ROS and clarified the roles of Schottky junctions, piezoelectric/plasmonic effects, and cavitation–photocatalytic coupling, quantitative structure–activity relationships remain limited. Systematic variation of MXene composition, surface terminations, and heterostructure architecture, supported by density functional theory and multi-physics cavitation modeling, will be essential to rationally design catalysts with tailored band structures, hydrophobicity, and ROS selectivity. Most existing studies focus on single contaminants and idealized water matrices. Future work should therefore prioritize real water systems, including surface waters, wastewater effluents, and industrial wastewater, where competitive adsorption effects and radical scavenging by coexisting species significantly influence performance. Future studies should include electrical energy per order under defined operating conditions, quantify energy yield based on pollutant removal and preferably mineralization, and assess cavitation efficiency considering acoustic power, reactor design, frequency, catalyst loading, and water chemistry. Economic analyses should include electricity use, catalyst synthesis/recovery, operation, maintenance, scale-up, and waste management to determine whether MXene-enhanced degradation justifies its energy and material costs.

**Fig. 7:**
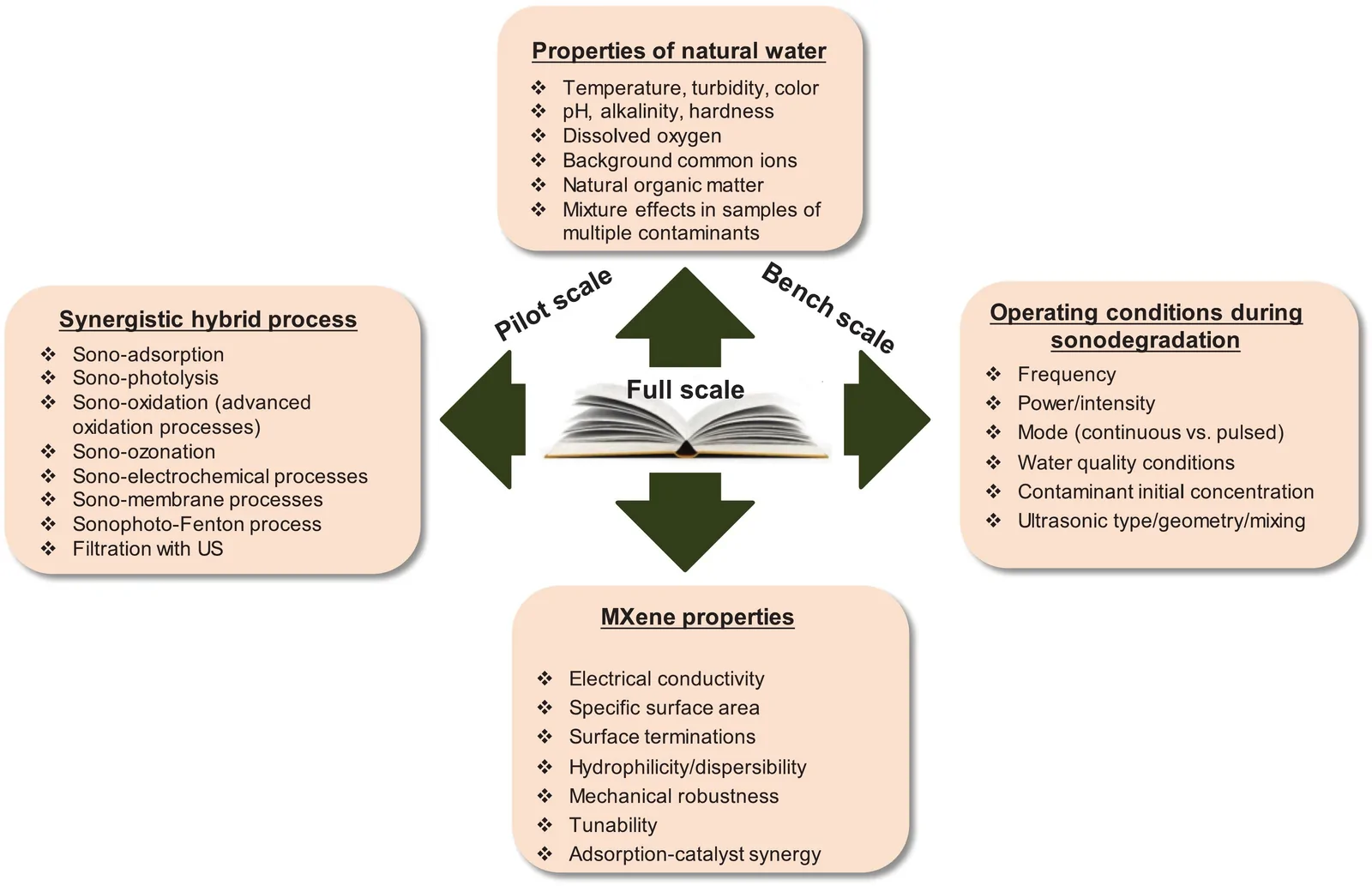
Potential areas of future study and key challenges in MXene-based sonocatalytic systems for water purification modified from [24].

Long-term stability, metal leaching, and by-product toxicity must also be rigorously evaluated using TOC analysis, inorganic ion balance, and bioassays. These assessments should be complemented by life-cycle and techno-economic analyses to benchmark MXene-based systems against established advanced oxidation processes. From a process engineering perspective, optimization of ultrasound frequency, power, and reactor geometry for MXene suspensions remains underexplored. Scale-up efforts should focus on energy-efficient reactor designs that ensure uniform cavitation, enable facile catalyst recovery, and are compatible with hybrid UV/visible-light and (sono-)Fenton systems. Finally, emerging research directions include: (i) designing selective transformation pathways that minimize toxic intermediates and enable partial oxidation into value-added or reusable products, and (ii) coupling sonocatalysis with renewable energy sources (e.g., solar irradiation and waste heat) alongside smart process control strategies, including in situ monitoring of H_2_O_2_ and ROS dynamics. Addressing these challenges will be critical for translating MXene-based sonocatalysis from laboratory-scale demonstrations into robust and sustainable water treatment technologies. The prospects, future research directions, and key challenges associated with sustainable ultrasonic water treatment, as synthesized in this review, are summarized in Fig. 7.

## 6 Conclusions

MXene-based sonocatalysis has emerged as a highly promising advanced oxidation technology for the removal of dyes, endocrine-disrupting compounds, and pharmaceuticals from water. Across a broad range of MXene compositions and heterostructures, contaminant removal is predominantly governed by ROS-driven oxidation, primarily involving ^•^OH with significant contributions from O_2_^•−^, while adsorption plays a secondary but system-dependent role influenced by contaminant speciation and MXene surface chemistry. This review demonstrates that water quality and operational parameters including pH, temperature, background ions and NOM, as well as ultrasound frequency and power critically govern cavitation dynamics, ROS generation, and degradation pathways. Near-neutral pH and moderate temperatures generally maximize oxidative degradation efficiency, whereas NOM and certain inorganic anions act as effective radical scavengers, thereby inhibiting performance.

The incorporation of radical promoters (e.g., H_2_O_2_, PMS, and PDS) and the use of hybrid systems such as UV/visible-light coupling and sono-Fenton processes significantly enhance ROS production and mineralization efficiency. These effects are particularly pronounced when MXenes are integrated into Schottky junctions, Z-scheme architectures, or piezo-plasmonic heterostructures, which improve charge separation and interfacial electron transfer. Mechanistic investigations employing radical scavengers, ESR, electrochemical analyses, and liquid chromatography–mass spectrometry consistently demonstrate that MXenes function as efficient electron mediators, cavitation nuclei, and interfacial engineering platforms. These features enhance charge separation and direct ROS generation toward contaminant mineralization pathways. TOC analyses further confirm that MXene-based sonocatalysts achieve not only parent compound degradation but also substantial mineralization. Stability and reusability studies across powders, membranes, and composite systems indicate that many MXene-based catalysts retain structural integrity, maintain high removal efficiencies (>80–90%), and exhibit minimal metal leaching over multiple cycles, supporting their applicability for practical use.

Overall, MXene-based materials provide a versatile and robust platform for tunable, high-efficiency sonocatalytic and sonophotocatalytic water treatment. However, future research must focus on mechanistic catalyst design, validation under realistic water conditions, reactor scale-up, and standardized performance evaluation to enable translation from laboratory research to full-scale environmental remediation technologies.

## CRediT authorship contribution statement

**Hak-Hyeon Kim:** Writing – original draft. **Seong-Nam Nam:** Writing – original draft. **Chang Min Park:** Writing – review & editing. **Min Jang:** Writing – review & editing. **Shane A. Snyder:** Writing – review & editing. **Byung-Moon Jun:** Writing – review & editing, Supervision, Project administration, Funding acquisition. **Yeomin Yoon:** Writing – review & editing, Supervision, Project administration, Funding acquisition.

## Declaration of competing interest

The authors declare that they have no known competing financial interests or personal relationships that could have appeared to influence the work reported in this paper.

## Data Availability

The datasets used or analyzed during the current study are available from the corresponding author upon reasonable request.
