# Supplementary material for: A review of MXene-based sonocatalytic degradation for water purification
Source: Ultrason Sonochem. 2026 Aug 22;133:108018. doi: 10.1016/j.ultsonch.2026.108018 (PMC13524752; doi:10.1016/j.ultsonch.2026.108018)
Supplement: Supplementary file 1 [file mmc1.docx]

*Supplementary Information for*

**A review of MXene-based sonocatalytic degradation for water purification**

Hak-Hyeon Kim^a, 1^, Seong-Nam Nam^b, 1^, Chang Min Park^c^, Min Jang^d^, Shane A. Snyder^a, e^,

Byung-Moon Jun^f, *^, Yeomin Yoon^a, **^

*^a^Department of Environmental Science & Engineering, Ewha Womans University,*

*52 Ewhayeodae-gil, Seodaemun-gu, Seoul 03760, Republic of Korea*

*^b^Department of Chemical and Environmental Science, Korea Army Academy at Yeongcheon, 495 Hoguk-ro, Gogyeong-myeon, Yeongcheon-si, Gyeongsangbuk-do, 38900, Republic of Korea*

*^c^Department of Environmental Engineering, Kyungpook National University, 80 Daehak-ro, Buk-gu, Daegu 41566, Republic of Korea*

*^d^Department of Environmental Engineering, Kwangwoon University, 447-1 Wolgye-dong Nowon-gu, Seoul, Republic of Korea*

*^e^School of Civil and Environmental Engineering, College of Engineering, Georgia Institute of Technology, 311 Ferst Dr NW, Atlanta, GA 30332, United States*

*^f^Department of Environmental Science and Engineering, Kyung Hee University,*

*1732 Deogyeong-daero, Giheung-gu, Yongin-si, Gyeonggi-do 17104, Republic of Korea*

**Corresponding author. E-mail:* [*jbm9101@khu.ac.kr*](mailto:jbm9101@khu.ac.kr) *(B. Jun)*

***Corresponding author. E-mail:* [*yoony@ewha.ac.kr*](mailto:yoony@ewha.ac.kr) *(Y. Yoon)*

^1^Hak-Hyeon Kim and Seong-Nam Nam contributed equally to this work.

**Table S1.** Summary of selected EDC and PhAC removal by US treatment.

| EDC/PPCP class | C_0_  (μg L^-1^) | Catalysts or  hybrids | Experimental conditions | Key removal or  rate constant | Ref. |  |
| --- | --- | --- | --- | --- | --- | --- |
| Acetaminophen (paracetamol) | 3,000-10,000 | Fe^2+^Fe_2_^3+^O_4_  (NADC-ECP) | SDW  37/80 kHz  36.36 W L^-1^  90 min | 13.8% (US)  32.6% (NADC-ECP)  60.8% (US/NADC-ECP) | [1] |  |
|  | 3,000 | Graphene oxide/β-Bi_2_O_3_/TiO_2_/Bi_2_Ti_2_O_7_ | SDW  28/580/970 kHz  180 W L^-1^  60 min | >99% | [2] |  |
|  | 10,000 | ZnO/  powdered stone waste | SDW  80 kHz  256 W  60 min | 2.27×10^-2^ min^-1^ | [3] |  |
|  | 5,000 | Fe-doped TiO_2_  H_2_O_2_ | SDW  50 kHz  296 W  30 min | >99% | [4] |  |
| Diclofenac | 1500 | Ti_3_C_2_Tx | SDW  28/570 kHz  180 W L^-1^  60 min | 0.040/0.072 min^-1^  (28/570 kHz)  (US)  0.071/0.080 min^-1^  (28/570 kHz)  (US+SLM)  0.069/0.089 min^-1^  (28/570 kHz)  (US+MLM) | [5] |  |
|  | 10,000 | PAC  UF | WWE  35/130 kHz  35/29 W L^-1^  30/60/120/180/240 min | >99% | [6] |  |
|  | 0.77 | Sono-photo-Fenton  Oxalic acid | WWE  375 kHz  88 W L^-1^  90 min | 22.1% (US/Fe)  13.0% (US/UV/Fe)  48.1% (US/UV/Fe/OA) | [7] |  |
| Ibuprofen | 20,000 | Sono-Fenton | SDW  12-862 kHz  25-100 W L ^-1^  180 min | 90% (sonolysis)  >99% (sono-Fenton) | [8] |  |
|  | 50,000 | Sea-mud catalyst (sea sludge dried in furnace 400℃ during 3 hr)  UV  H_2_O_2_ | PWW  37 kHz  2.13 W mL ^-1^ (effective power: 0.53 W mL ^-1^)  60 min | 0.042 min^-1^ (continuous US)  0.04 min^-1^  (pulsed US) | [9] |  |
|  | 100,000 | Zeolite-titanate  UV  H_2_O_2_ | SDW  37 kHz  0.26 W mL ^-1^  100 min | 0.0302 min^-1^  (UV/zeolite-TiO_2_/H_2_O_2_/US)  0.0096 min^-1^  (UV/zeolite-TiO_2_/H_2_O_2_) | [10] |  |
|  | 10,000 | Zero-valent iron | SDW  20-1130 kHz  67-600 W L^-1^  30 min | 0.067/0.090/0.095/0.280/0.135 min^-1^  (20/40/200/572/1130 kHz) | [11] |  |
| Salicylic acid | 2,800 | Metal organic framework | SDW  28/1000 kHz  45-180 W L^-1^  60 min | 46/60%  (28/1000 kHz)  (w/MOF)  20/37%  (28/1000 kHz)  (w/o MOF) | [12] |  |
| Verapamil | 2300 | Ti_3_C_2_T_x_ | SDW  28/570 kHz  180 W L^-1^  60 min | 0.040/0.072 min^-1^  (28/570 kHz)  (US)  0.071/0.101 min^-1^  (28/570 kHz)  (US+SLM)  0.077/0.128 min^-1^  (28/570 kHz)  (US+MLM) | [5] |  |
| Amoxicillin | 10,000 | PAC  UF | WWE  35/130 kHz  35/29 W L^-1^  30/60/120/180/240 min | >99% | [6] |  |
|  | 25,000 | O_3_ | SDW  575/861/1141 kHz  14.6 W L^-1^  90 min | 0.04 min^-1^  (US)  1.97 min^-1^  (O_3_)  2.5 min^-1^  (US/O_3_) | [13] |  |
| Ampicillin | 10,500 | Sono-photo-Fenton | SDW  375 kHz  0.0976 W mL^-1^  60 min | ~80%(US/Fe)  >99%(US/UV/Fe) | [14] |  |
| Cefixime | 100,000-200,000 | Sono-photo (US/UV/ZnO) | SDW  40 kHz  4.76 W L^-1^  100-160 min | 51.5-73.4%  (200,000-100,000 μg L^-1^) | [15] |  |
|  | 10,400 | Sono-electro-Fenton | SWW  37 kHz  15-90 min | 0.0253 min^-1^ | [16] |  |
| Ciprofloxacin | 8,000 | PMS  ZnO | SDW  60 kHz  550 W  120 min | 98.3% | [17] |  |
| Flumequine | 260,000 | Sono-Fenton | SDW  40 kHz  120 W  120 min | 73% (Fenton)  93% (Sono-Fenton) | [18] |  |
| Metronidazole | 10,000 | TiO_2_/Fe_3_O_4_ | SDW  20 kHz  40 W  90 min | 97.5% | [19] |  |
| Penicillin G | 100,000-200,000 | Sono-photo  (US/UV/ZnO) | SDW  40 kHz  4.76 W L^-1^  100-160 min | 30.7-63.3%  (200,000-100,000 μg L^-1^) | [15] |  |
| Rifampin | 15,000 | Cr_2_AlC  H_2_O_2_ | SDW  36 kHz  150 W L^-1^  120 min | 94.5% | [20] |  |
|  | 30,000 | N-doped ZnO/GO | SDW  400 W  60 min | 0.0005 min^-1^  (US)  0.02 min^-1^  (US+GO/ZnO)  0.0466 min^-1^  (US+N:ZnO/GO)  0.036 min^-1^  (N:ZnO) | [21] |  |
| Sulfadiazine | 40,000 | Magnetic graphene oxide−polydopamine | SDW  40 kHz  150/300/450 W  60 min | 0.0013 min^-1^ (US)  0.0094 min^-1^  (US/Fe_3_O_4_)  0.011 min^-1^  (US/GO)  0.0273 min^-1^  ^(^US/GO-PDA)  0.0399  (US/M-GO-PDA) | [22] |  |
| Sulfamethoxazole |  | TiO_2_  UV | SDW  35 kHz  120 min | 3.0×10^-3^ min^-1^ (US)  1.62×10^-2^ min^-1^ (UV/TiO_2_)  6.37×10^-2^ min^-1^ (US/UV/TiO_2_) | [23] |  |
|  |  | TiO_2_  UV | WWE  35 kHz  120 min | 5.87×10^-3^ min^-1^ (UV/TiO_2_)  4.5×10^-2^ min^-1^ (US/UV/TiO_2_) | [23] |  |
| Tetracycline | 44,000 | Reduced graphene oxide (rGO), PMS | SDW  35 kHz  240 W  30 min |  | [24] |  |
|  | 89,000 | Ag@LiNbO_3_/PVDF | SDW  40 kHz  120 W  120 min | 8.87×10^-3^ min^-1^ | [25] |  |
|  | 20,000 | Cu_2_O/BiFeO_3_ | SDW  40 Hz  500 W  120 min | 83.5/98.0% (100/500 W)  0.00129 min^-1^ (US)  0.00515 min^-1^ (US+BiFeO_3_)  0.02302 min^-1^ (US+CBF-3) | [26] |  |
|  | 15,000-70,000 | PMS  CeO_2_ | SDW  20 kHz  40/50/70 W  120 min | 12.3% (US)  40.1% (US+PMS)  58.0% (US+CeO_2_)  92.6%  (US+PMS+CeO_2_) | [27] |  |
|  |  |  |  |  |  |  |
| Carbamazepine | 4,700 | Metal organic frameworks | SDW  28/1000 kHz  45-180 W L^-1^  60 min | 47/99%  (28/1000 kHz)  (w/MOF)  25/97%  (28/1000 kHz)  (w/o MOF) | [12] |  |
|  | 10,000 | PAC  UF | WWE  35/130 kHz  35/29 W L^-1^  30/60/120/180/240 min | >99% | [6] |  |
|  | 4,700 | Graphene oxide/β-Bi_2_O_3_/TiO_2_/Bi_2_Ti_2_O_7_ | SDW  28/580/970 kHz  180 W L^-1^  60 min | >99% | [2] |  |
| Hydroxychloroquine | 15,000 | Cr_2_AlC, H_2_O_2_ | SDW  36 kHz  150 W L^-1^  120 min | >99% | [20] |  |
| 17β-Estradiol (E2) | 2,000 | Na_2_S_2_O_8_ | SDW  20 kHz  88/154/212 W L^-1^  15, 30, 45, 60, 90 min | 0.0092/0.0121/0.0173 min^-1^  (88/154/212 W L^-1^)  (w/o Na_2_S_2_O_8_)  0.0332 min^-1^  (w/Na_2_S_2_O_8_) | [28] |  |
| Atrazine |  | TiO_2_  UV | SDW  35 kHz  120 min | 1.60×10^-2^ min^-1^ (US)  4.80×10^-2^ min^-1^ (UV/TiO_2_)  8.67×10^-2^ min^-1^ (US/UV/TiO_2_) | [23] |  |
|  |  | TiO_2_  UV | WWE  35 kHz  120 min | 1.4×10^-2^ min^-1^ (UV/TiO_2_)  3.9×10^-2^ min^-1^ (US/UV/TiO_2_) | [23] |  |
| 4-chlorophenol/ | 10,000-100,000 | Persulfate,  nZVC | SDW  40 kHz  70 W L^-1^  60 min | 31%(US/nZVC)  75%(US/PS)  98%(US/PS/nZVC) | [29] |  |
| Bisphenol A | 23,000 | Reduced graphene oxide (rGO), PMS | SDW  35 kHz  240 W  40 min |  | [24] |  |
|  | 1,000 | UV | NSW  20 kHz  9/17/26/43 W cm^-2^  30 min | 0.053/0.10/0.213/0.320 min^-1^  (9/17/26/43 W cm^-2^)  (US)  0.401 min^-1^  (43 W cm^-2^)  (UV/US) | [30] |  |
|  | 1,000 | Sonoelectrochemical hybrid system | SWW  24 kHz  0.4 W mL^-1^  30 min | 0.024 min^-1^  (AC)  0.016 min^-1^  (EC, (5 V))  0.090 min^-1^  (AC/EC (5 V)) | [31] |  |
|  | 15,000 | Sono-photo-Fenton;  Perovskite  (LaFeO_3_) | SWW  20 kHz  40 W L^-1^  180 min | 13.5/17% (US/US+H_2_O_2_)  19.9% (LaFeO_3_+H_2_O_2_+US)  20.8% (LaFeO_3_+ US+visible light)  21.4% (LaFeO_3_+US)  21.8% (LaFeO_3_+ US+visible light+H_2_O_2_) | [32] |  |
|  | 23,000 | Manganese dioxide,  biochar | SWW  20 kHz  0.26 W mL^-1^  120 min | 0.0008 min^-1^  (US)  0.0021 min^-1^  (US+H_2_O_2_)  0.0082 min^-1^  (US+ α-MnO_2_/BCs)  0.1565 min^-1^  (US+ α-MnO_2_/BCs  +H_2_O_2_) | [33] |  |
|  | 5,000 | Persulfate,  mechanical mixing (400 rpm) | SWW  35 kHz  76 W L^-1^ | 4.20×10^-3^ min^-1^  (US/M)  5.86×10^-3^ min^-1^  (US/M-PS) | [34] |  |
|  |  | TiO_2_  UV | SDW  35 kHz  120 min | 3.0×10^-2^ min^-1^ (US)  3.68×10^-2^ min^-1^ (UV/TiO_2_)  8.75×10^-2^ min^-1^ (US/UV/TiO_2_) | [23] |  |
|  |  | TiO_2_  UV | WWE  35 kHz  120 min | 1.3×10^-2^ min^-1^ (UV/TiO_2_)  4.4×10^-2^ min^-1^ (US/UV/TiO_2_) | [23] |  |
|  | 10,000 | Sono-photo  (Ni_x_Mg_4-x_S_4_@  Ti_3_C_2_T_x_)  (visible light) | SDW  970 kHz  34 W L^-1^  60 min | 38.1% (NMO)  61.3% (NMS)  91.6% (NMS@MX) | [35] |  |
|  | 2,300, | PMS | SDW  20/200/400 kHz  0.14/0.09/0.11 W mL^-1^  30 min | 0.04/0.051/0.063 min^-1^  (20/200/400 kHz)  (w/o PMS)  0.051/0.085/0.140 min^-1^  (20/200/400 kHz)  (w/ PMS) | [36] |  |
| Dimethyl phthalate | 15,000 | Cr_2_AlC, H_2_O_2_ | SWW  36 kHz  150 W L^-1^  120 min | 10.8% (US)  37.5% (H_2_O_2_/US)  44.6% (Cr_2_AlC/US)  69.1% (Cr_2_AlC/H_2_O_2_/US) | [20] |  |
|  | 1,900 | PMS | SDW  20/200/400 kHz  0.14/0.09/0.11 W mL^-1^  30 min | 0.003/0.023/0.030 min^-1^  (20/200/400 kHz)  (w/o PMS)  0.005/0.050/0.063  min^-1^  (20/200/400 kHz)  (w/ PMS) | [36] |  |
| Tris-2-chloroethyl phosphate | 2,000 | Na_2_S_2_O_8_ | SWW  20 kHz  88/154/212 W L^-1^  15, 30, 45, 60, 90 min | 0.0019/0.0028/0.0035 min-1  (88/154/212 W L-1)  (US)  0.0068 min^-1^  (US/Na_2_S_2_O_8_) | [28] |  |
| Pyrene | 1,000 | UV | NSW  20 kHz  9/17/26/43 W cm^-2^  30 min | 0.018/0.036/0.052  /0.21 min^-1^  (9/17/26/43 W cm^-2^, respectively)  (US)  0.23 min^-1^  (43 W cm^-2^)  (UV/US) | [30] |  |
| Methylparaben | 5,000-60,000 | Sepiolite | SDW  572/861 kHz  0.19 W L^-1^  10-30 min | 0.098/0.090/0.068/0.054/0.040/0.024 min^-1^  (5/10/15/20/30/60×10^3^ μg L^-1^)  (w/o sepiolite)  0.076-0.095 min^-1^  (w/ sepiolite) | [37] |  |
| Gemfibrozil | 2,000 | Na_2_S_2_O_8_ | SWW  20 kHz  88/154/212 W L^-1^  15/30/45/60/90 min | 0.0109/0.0152/0.0195 min^-1^  (88/154/212 W L^-1^)  (US)  0.0388 min^-1^  (US/Na_2_S_2_O_8_) | [28] |  |

Modified from [38]:

AC = acoustic cavitation; BC = biochar; C_0_ = EDC or PPCP initial concentration; CBF-3 = Cu_2_O/BiFeO_3_; CNTs = carbon nanotubes; COD = chemical oxygen demand; EC = electrochemical degradation; GO = graphene oxide; M = mechanical mixing; MLM = multilayered MXene; MOF = metal organic framework; MX = Ti_3_C_2_T_x_ MXene; NADC-ECP = nano-composite air-dispersion cathode; NMO = nickel magnesium oxide; NMS = Ni_x_Mg_4-x_S_4_; NSW = natural surface water; nZVC = nanoscale zero-valent copper; OA = oxalic acid; PAC = powdered activated carbon; PDA = polydopamine; PMS = peroxymonosulfate; PS = persulfate; PVDF = polyvinylidene difluoride; PWW = pharmaceutical wastewater; SDW = synthetic drinking water; SLM = single layered MXene; SSW = synthetic seawater; SWW = synthetic wastewater; UF = ultrafiltation membrane; US = ultrasonic; UV = ultraviolet; w/ = with; w/o = without; WWE = wastewater effluent.

**References**

[1] R. Mirzaee, R.D.C. Soltani, A. Khataee, G. Boczkaj, , Combination of air-dispersion cathode with sacrificial iron anode generating Fe2+Fe23+O4 nanostructures to degrade paracetamol under ultrasonic irradiation, Journal of Molecular Liquids 284 (2019) 536-546.

[2] G. Lee, K.H. Chu, Y.A.J. Al-Hamadani, C.M. Park, M. Jang, J. Heo, N. Her, D.H. Kim, Y. Yoon, , Fabrication of graphene-oxide/beta-Bi2O3/TiO2/Bi2Ti2O7 heterojuncted nanocomposite and its sonocatalytic degradation for selected pharmaceuticals, Chemosphere 212 (2018) 723-733.

[3] R.D.C. Soltani, Z. Miraftabi, M. Mahmoudi, S. Jorfi, G. Boczkaj, A. Khataee, , Stone cutting industry waste-supported zinc oxide nanostructures for ultrasonic assisted decomposition of an anti-inflammatory non-steroidal pharmaceutical compound, Ultrasonics Sonochemistry 58 (2019) 104669.

[4] H.C. Yap, Y.L. Pang, S. Lim, C.W. Lai, A.Z. Abdullah, , Enhanced sonophotocatalytic degradation of paracetamol in the presence of Fe-doped TiO2 nanoparticles and H2O2, Environmental Earth Sciences 79(19) (2020) 457.

[5] S. Kim, S.N. Nam, C.M. Park, M. Jang, N. Taheri-Qazvini, Y. Yoon, , Effect of single and multilayered Ti3C2TX MXene as a catalyst and adsorbent on enhanced sonodegradation of diclofenac and verapamil, Journal of Hazardous Materials 426 (2022) 128120.

[6] V. Naddeo, M.F.N. Secondes, L. Borea, S.W. Hasan, F. Ballesteros, V. Belgiorno, , Removal of contaminants of emerging concern from real wastewater by an innovative hybrid membrane process - UltraSound, Adsorption, and Membrane ultrafiltration (USAMe (R)), Ultrasonics Sonochemistry 68 (2020) 105237.

[7] E.A. Serna-Galvis, A.M. Botero-Coy, D. Martinez-Pachon, A. Moncayo-Lasso, M. Ibanez, F. Hernandez, R.A. Torres-Palma, , Degradation of seventeen contaminants of emerging concern in municipal wastewater effluents by sonochemical advanced oxidation processes, Water Research 154 (2019) 349-360.

[8] S. Adityosulindro, L. Barthe, K. Gonzalez-Labrada, U.J.J. Haza, H. Delmas, C. Julcour, , Sonolysis and sono-Fenton oxidation for removal of ibuprofen in (waste) water, Ultrasonics Sonochemistry 39 (2017) 889-896.

[9] Z. Asadi, S. Dobaradaran, H. Arfaeinia, M. Omidvar, S. Farjadfard, R. Foroutan, B. Ramavandi, R. Luque, , Photodegradation of ibuprofen laden-wastewater using sea-mud catalyst/H2O2 system: evaluation of sonication modes and energy consumption, Environmental Science and Pollution Research (2022) 16707-16718.

[10] N. Farhadi, T. Tabatabaie, B. Ramavandi, F. Amiri, , Optimization and characterization of zeolite-titanate for ibuprofen elimination by sonication/hydrogen peroxide/ultraviolet activity, Ultrasonics Sonochemistry 67 (2020) 105122.

[11] A. Ziylan-Yavas, N.H. Ince, , Single, simultaneous and sequential applications of ultrasonic frequencies for the elimination of ibuprofen in water, Ultrasonics Sonochemistry 40 (2018) 17-23.

[12] B.M. Jun, S. Kim, J. Heo, N. Her, M. Jang, C.M. Park, Y. Yoon, , Enhanced sonocatalytic degradation of carbamazepine and salicylic acid using a metal-organic framework, Ultrasonics Sonochemistry 56 (2019) 174-182.

[13] R. Kidak, S. Dogan, , Medium-high frequency ultrasound and ozone based advanced oxidation for amoxicillin removal in water, Ultrasonics Sonochemistry 40 (2018) 131-139.

[14] D.M. Montoya-Rodriguez, E.A. Serna-Galvis, F. Ferraro, R.A. Torres-Palma, , Degradation of the emerging concern pollutant ampicillin in aqueous media by sonochemical advanced oxidation processes - Parameters effect, removal of antimicrobial activity and pollutant treatment in hydrolyzed urine, Journal of Environmental Management 261 (2020) 110224.

[15] A. Almasi, M. Mohammadi, F. Baniamerian, Z. Berizi, M.H. Almasi, Z. Pariz, , Modeling of antibiotic degradation in sonophotocatalytic process, increasing biodegradability and process optimization by response surface methodology (RSM), International Journal of Environmental Science and Technology 16(12) (2019) 8437-8448.

[16] K. Hasani, A. Peyghami, A. Moharrami, M. Vosoughi, A. Dargahi, , The efficacy of sono-electro-fenton process for removal of cefixime antibiotic from aqueous solutions by response surface methodology (RSM) and evaluation of toxicity of effluent by microorganisms, Arabian Journal of Chemistry 13(7) (2020) 6122-6139.

[17] N. Firouzeh, M. Malakootian, S.N. Asadzadeh, M. Khatami, Z. Makarem, , Degradation of ciprofloxacin using ultrasound/ZnO/Oxone process from aqueous solution-lab-scale analysis and optimization, Bionanoscience 11(2) (2021) 306-313.

[18] M. Harrabi, H.B. Ammar, K. Mbarki, I. Naifar, C. Yaiche, F. Aloulou, B. Elleuch, , Ultrasonic power improvement of flumequine degradation effectiveness in aqueous solution via direct and indirect action of mechanical acoustic wave, Ultrasonics Sonochemistry 48 (2018) 517-522.

[19] A. Sheikhmohammadi, E. Asgari, H. Nourmoradi, M.M. Fazli, M. Yeganeh, , Ultrasound-assisted decomposition of metronidazole by synthesized TiO2/Fe3O4 nanocatalyst: Influencing factors and mechanisms, Journal of Environmental Chemical Engineering 9(5) (2021) 105844.

[20] M. Alimohamadi, A. Khataee, S. Arefi-Oskoui, B. Vahid, Y. Orooji, Y. Yoon, , Catalytic activation of hydrogen peroxide by Cr2AlC MAX phase under ultrasound waves for a treatment of water contaminated with organic pollutants, Ultrasonics Sonochemistry 93 (2023) 106294.

[21] A.A. Bazghale, A. Mohammad-Khah, , Improvement of ultrasound-assisted removal of rifampin in the presence of N: ZnO/GO nanocomposite as sonocatalyst, Chemistryselect 5(15) (2020) 4413-4421.

[22] P. Gholami, A. Khataee, B. Vahid, , Integration of polydopamine and Fe3O4 nanoparticles with graphene oxide to fabricate an efficient recoverable catalyst for the degradation of sulfadiazine, Industrial & Engineering Chemistry Research 59(1) (2020) 183-193.

[23] B. Ryu, K.T. Wong, C.E. Choong, J.R. Kim, H. Kim, S.H. Kim, B.H. Jeon, Y. Yoon, S.A. Snyder, M. Jang, , Degradation synergism between sonolysis and photocatalysis for organic pollutants with different hydrophobicity: A perspective of mechanism and application for high mineralization efficiency, Journal of Hazardous Materials 416 (2021) 125787.

[24] Y. Cherifi, A. Addad, H. Vezin, A. Barras, B. Ouddane, A. Chaouchi, S. Szunerits, R. Boukherroub, , PMS activation using reduced graphene oxide under sonication: Efficient metal-free catalytic system for the degradation of rhodamine B, bisphenol A, and tetracycline, Ultrasonics Sonochemistry 52 (2019) 164-175.

[25] G. Singh, M. Sharma, R. Vaish, , Flexible Ag@LiNbO3/PVDF composite film for piezocatalytic dye/pharmaceutical degradation and bacterial disinfection, Acs Applied Materials & Interfaces 13(19) (2021) 22914-22925.

[26] X. Wang, X.S. He, C.Y. Li, S.L. Liu, W. Lu, Z. Xiang, Y. Wang, , Sonocatalytic removal of tetracycline in the presence of S-scheme Cu2O/ BiFeO3 heterojunction: Operating parameters, mechanisms, degradation pathways and toxicological evaluation, Journal of Water Process Engineering 51 (2023) 103345.

[27] S.N. Asadzadeh, M. Malakootian, M. Mehdipoor, D.K. Neyestanaki, , The removal of tetracycline with biogenic CeO2 nanoparticles in combination with US/PMS process from aqueous solutions: kinetics and mechanism, Water Science and Technology 83(6) (2021) 1470-1482.

[28] J.R.A. Corena, J.A. Bergendahl, , Effect of pH, temperature, and use of synergistic oxidative agents on the ultrasonic degradation of tris-2-chloroethyl phosphate, gemfibrozil, and 17 beta estradiol in water, Journal of Environmental Chemical Engineering 9(1) (2021) 105005.

[29] Z. Sharifi, G. Asgari, A. Seid-mohammadi, , Sonocatalytic Degradation of p-Chlorophenol by Nanoscale Zero-valent Copper Activated Persulfate under Ultrasonic Irradiation in Aqueous Solutions, International Journal of Engineering 33(6) (2020) 1061-1069.

[30] J. Copik, E. Kudlek, M. Dudziak, , Degradation of bisphenol A and pyrene from highway retention basin water using ultrasound enhanced by UV irradiation, Architecture Civil Engineering Environment 15(2) (2022) 135-148.

[31] M. Dietrich, M. Franke, M. Stelter, P. Braeutigam, , Degradation of endocrine disruptor bisphenol A by ultrasound-assisted electrochemical oxidation in water, Ultrasonics Sonochemistry 39 (2017) 741-749.

[32] M. Dukkanci, , Sono-photo-fenton oxidation of bisphenol-A over a LaFeO3 perovskite catalyst, Ultrasonics Sonochemistry 40 (2018) 110-116.

[33] K.W. Jung, S.Y. Lee, Y.J. Lee, J.W. Choi, , Ultrasound-assisted heterogeneous Fenton-like process for bisphenol A removal at neutral pH using hierarchically structured manganese dioxide/biochar nanocomposites as catalysts, Ultrasonics Sonochemistry 57 (2019) 22-28.

[34] Y. Lee, S. Lee, M. Cui, J. Kim, J. Ma, Z. Han, J. Khim, , Improving sono-activated persulfate oxidation using mechanical mixing in a 35-kHz ultrasonic reactor: Persulfate activation mechanism and its application, Ultrasonics Sonochemistry 72 (2021) 105412.

[35] S. Vigneshwaran, B.M. Jun, S.M. Prabhu, S.S. Elanchezhiyan, Y.S. Ok, S. Meenakshi, C.M. Park, , Enhanced sonophotocatalytic degradation of bisphenol A using bimetal sulfide-intercalated MXenes, 2D/2D nanocomposite, Separation and Purification Technology 250 (2020) 117178.

[36] L.J. Xu, X.T. Wang, Y. Sun, H. Gong, M.Z. Guo, X.M. Zhang, L. Meng, L. Gan, , Mechanistic study on the combination of ultrasound and peroxymonosulfate for the decomposition of endocrine disrupting compounds, Ultrasonics Sonochemistry 60 (2020) 104749.

[37] B. Savun-Hekimoglu, N.H. Ince, , Sonochemical and sonocatalytic destruction of methylparaben using raw, modified and SDS-intercalated particles of a natural clay mineral, Ultrasonics Sonochemistry 54 (2019) 233-240.

[38] S.J. An, S.N. Nam, J.S. Choi, C.M. Park, M. Jang, J.Y. Lee, B.M. Jun, Y. Yoon, Ultrasonic treatment of endocrine disrupting compounds, pharmaceuticals, and personal care products in water: An updated review, J. Hazard. Mater. 474 (2024) 134852.
